# Genotype-Specific Expression and NLR Repertoire Contribute to Phenotypic Resistance Diversity in *Plantago lanceolata*

**DOI:** 10.3389/fpls.2021.675760

**Published:** 2021-07-12

**Authors:** Pezhman Safdari, Layla Höckerstedt, Mikael Brosche, Jarkko Salojärvi, Anna-Liisa Laine

**Affiliations:** ^1^Organismal and Evolutionary Biology Research Programme, University of Helsinki, Helsinki, Finland; ^2^Climate System Research, Finnish Meteorological Institute, Helsinki, Finland; ^3^School of Biological Sciences, Nanyang Technological University, Singapore, Singapore; ^4^Department of Evolutionary Biology and Environmental Studies, University of Zurich, Zurich, Switzerland

**Keywords:** phenotypic variation, pathogen-imposed selection, expression profile, phenotypic resistance diversity, natural host populations

## Abstract

High levels of phenotypic variation in resistance appears to be nearly ubiquitous across natural host populations. Molecular processes contributing to this variation in nature are still poorly known, although theory predicts resistance to evolve at specific loci driven by pathogen-imposed selection. Nucleotide-binding leucine-rich repeat (NLR) genes play an important role in pathogen recognition, downstream defense responses and defense signaling. Identifying the natural variation in NLRs has the potential to increase our understanding of how NLR diversity is generated and maintained, and how to manage disease resistance. Here, we sequenced the transcriptomes of five different *Plantago lanceolata* genotypes when inoculated by the same strain of obligate fungal pathogen *Podosphaera plantaginis*. A *de novo* transcriptome assembly of RNA-sequencing data yielded 24,332 gene models with N50 value of 1,329 base pairs and gene space completeness of 66.5%. The gene expression data showed highly varying responses where each plant genotype demonstrated a unique expression profile in response to the pathogen, regardless of the resistance phenotype. Analysis on the conserved NB-ARC domain demonstrated a diverse NLR repertoire in *P. lanceolata* consistent with the high phenotypic resistance diversity in this species. We find evidence of selection generating diversity at some of the NLR loci. Jointly, our results demonstrate that phenotypic resistance diversity results from a crosstalk between different defense mechanisms. In conclusion, characterizing the architecture of resistance in natural host populations may shed unprecedented light on the potential of evolution to generate variation.

## Introduction

Parasitism is the most common life-style on Earth (Weinstein and Kuris, 2016), and parasitic species, including pathogens, play an important role in shaping biodiversity in natural populations (Kursar et al., 2009; Bever et al., 2015). Despite this, relatively little is still understood of the molecular mechanisms that enable hosts and parasites to coexist in natural populations. The threats imposed by pathogens on humans and on managed food production systems have motivated research that aims to predict where pathogens will occur and how risks of infection evolve (Koff, 1992; Woolhouse et al., 2001; Gilligan, 2002). In agriculture, increasing the diversity of crops – even from a monoculture to a mixture of two cultivars – has been shown to reduce disease levels (Zhu et al., 2000; Mundt, 2002). Meanwhile, natural host populations typically support diversity in resistance phenotypes (Salvaudon et al., 2008; Laine et al., 2011), and limited data available to date show that increasing resistance diversity decreases disease risk also in the wild (Jousimo et al., 2014). Thus, understanding how diversity in resistance is generated and maintained in natural populations can yield valuable insight into how to deploy durable resistance in crop plants.

Hosts and pathogens are assumed to coevolve through Red Queen dynamics, where the pathogen overcomes host’s defenses and the host in turn responds with new counter-defenses (Jaenike, 1978; Hamilton, 1980). Theory predicts such reciprocal coevolutionary selection to be a powerful mechanism for maintaining diversity in both host and parasite populations, as the selection rate for resistance depends on the frequency of parasite alleles, and vice versa, in a negative indirect frequency-dependent manner (Leonard, 1977; Bergelson et al., 2001). There are numerous examples of pathogens overcoming host resistance, both from agriculture and from the wild (Mundt, 2002, 2014). While evidence of resistance evolving under pathogen attack in the wild is scarce (Laine, 2006), there is ample support for coevolution from local adaptation studies where parasite/host fitness is measured in sympatry vs. allopatry (Greischar and Koskella, 2007; Hoeksema and Forde, 2008). To date, a handful of ground-breaking studies have demonstrated that fluctuations in resistance and infectivity in natural systems match the predictions of coevolutionary selection (Decaestecker et al., 2007; Gómez and Buckling, 2011; Thrall et al., 2012).

At molecular level, plant resistance is established through multi-layered defense mechanisms. First, there are physical defense barriers (Miedes et al., 2014) and overcoming these obstacles by a successful pathogen will trigger the pathogen-associated molecular patterns (PAMPs), which will trigger the so-called PAMP-triggered immunity (PTI) response, aimed at stopping the pathogen infection even before it begins. Third layer is the effector triggered immunity (ETI), involving either direct or indirect recognition of pathogen virulence factors (effector proteins; Jones and Dangl, 2006). In reality, as Thomma et al. (2011) point out, there is no clear line between different stages of the plant defense mechanism and PTI and ETI responses often overlap. Pathogen recognition involves a multitude of different signaling pathways, including production of reactive oxygen species, elevated Ca^2+^ and MAP kinases leading to activation of plant defenses. These defenses include the induction of stress hormones salicylic acid, jasmonic acid and ethylene, as well as extensive transcriptional re-programming ultimately resulting in the production of defensive compounds, such as antimicrobial secondary metabolites, chemicals and enzymes. As the final line of defense, plants may activate the hyper-sensitive response, programmed cell death, to rapidly kill the cells surrounding the infection, thus preventing the spread to nearby tissues (Coll et al., 2011; Egorov and Odintsova, 2012).

Many of the proteins involved in intracellular pathogen recognition belong to nucleotide-binding–leucine-rich repeat (NLR) protein family (Monteiro and Nishimura, 2018). They are involved both in direct and indirect recognition of the pathogen’s effector proteins, as well as in triggering the plant immune responses (Meunier and Broz, 2017). NLRs also contribute to signaling and transcript regulation (Chisholm et al., 2006; Jones and Dangl, 2006), and play an important role in local adaptation and habitat expansion of plants (Thrall et al., 2012; Stam et al., 2019). The antagonistic interaction between plant NLRs and pathogen effector proteins is considered to have a profound effect on the evolution of both organisms, shaping their genomes and gene repertoire (Upson et al., 2018). NLRs often form large tandemly arrayed gene families and hence questions regarding their origins and evolution have been under active research in both plants and animals (Borrelli et al., 2018; Andolfo et al., 2019). The numbers of identified NLRs differ substantially within and between plant families (Baggs et al., 2017), for example *Arabidopsis thaliana* (Arabidopsis) contains between 165 and 251 NLRs (Shao et al., 2016; Van de Weyer et al., 2019) and crop species such as wheat, barley, rice, tomato and potato contain 1,560, 224, 438, 137, and 309 NLRs, respectively (Sarris et al., 2016; Steuernagel et al., 2020). In *A. thaliana* there is evidence of widespread positive selection in the core NLRs shared among accessions, especially in the canonical NLR domains (Van de Weyer et al., 2019), while a pioneering work on wild tomato revealed high NLR diversity with a small subset of NLRs driving local adaptation to pathogens (Stam et al., 2016, 2019). Specific functions have been assigned to plant NLR domains. An NB-ARC domain is present in all full length NLRs and considered a regulatory domain (Takken et al., 2006) determining whether the protein is active or inactive (Takken and Goverse, 2012). Other canonical domains include Toll/interleukin-1 receptor (TIR), coiled coil (CC), and RPW8-like coiled-coil (CCR); their presence defines the sub-category of the NLR (TNL, CNL, or RNL, respectively) (Van de Weyer et al., 2019). Additionally, the NLRs contain several leucine-rich repeats (LRRs) which contribute to pathogen-specificity.

Here we study resistance in the ribwort plantain, *Plantago lanceolata* L. (Plantago), against its fungal pathogen *Podosphaera plantaginis*. Due to its global distribution, *P. lanceolata* has emerged as a model species to study how global change drives the ecology and evolution of natural plant populations (Jousimo et al., 2014; Smith et al., 2020). It is a perennial monecious plant that reproduces both sexually by wind pollination and clonally by producing side rosettes (Sagar and Harper, 1964). The powdery mildew, *P. plantaginis* (Castagne; U. Braun, and S. Takamatsu) (*Erysiphales*, Ascomycota), is a specialist obligate biotroph infecting *P. lanceolata.* It requires living host tissue throughout its life (Bushnell, 2002), and completes its life cycle as localized lesions on host leaves. The interaction between *P. lanceolata* and *P. plantaginis* depends on both host and pathogen genotypes suggesting gene-for-gene type of control (Thompson and Burdon, 1992; Laine, 2004, 2007). The putative resistance mechanism includes two steps, recognition of the attacking pathogen and then blocking its growth (Laine, 2004). In resistant phenotypes no pathogen growth is detected or the plant shows rapid cell death around infection site, whereas susceptible phenotypes demonstrate considerable variation in pathogen development, depending on both host and pathogen genotype (Laine, 2007). Previous studies have detected considerable phenotypic variation in resistance (Laine, 2004); diversity is shown to accumulate in the well-connected populations across the landscape (Hockerstedt et al., 2018), and has a direct negative impact on disease dynamics (Jousimo et al., 2014). Moreover, there is evidence of on-going coevolution in this interaction (Laine, 2005, 2006, 2008).

Here, we carried out a controlled experiment where five *P. lanceolata* genotypes were inoculated with the same *P. plantaginis* strain, in order to characterize the transcriptional responses and regulatory pathways activated in response to the inoculation. We assembled the first *de novo* transcriptome for Plantago to characterize the transcriptional responses in both resistant and susceptible phenotypes, and scanned the NLR repertoire for signs of selection. Since reliable *de novo* assembly of NLR transcripts is difficult due to highly repetitive nature of the LRR domains, we focused on the conserved NB-ARC domains in the evolutionary analyses. Each plant genotype demonstrated a unique gene expression profile in response to the pathogen, revealing a diverse NLR repertoire in Plantago, consistent with the high phenotypic resistance diversity uncovered in earlier studies.

## Materials and Methods

### Study System and Plant and Fungal Material

An inoculation protocol where conidia from small colonies or individual chains are placed on detached leaves or intact leaves of plants yields a robust characterization of resistance-susceptibility phenotype. From an earlier large inoculation study consisting of 2,944 host genotype–pathogen genotype combinations (Hockerstedt et al., 2018), we selected three genotypes (IDs 193_2, 2818_3 and 2818_6, named Res1, Res2, and Res3 hereafter) that were resistant against all tested pathogen strains, and two genotypes (IDs 313_6, 1553_5, named Sus1 and Sus2 hereafter) that were susceptible to all tested pathogen strains. For the experiment each genotype was cloned into six plants as described in Laine (2004).

### Inoculation Experiment

Two-month old plantlets (five genotypes with three replicates and two conditions, total of 30 plants) were inoculated with *P. plantaginis* strain Lammi_3 by brushing spores gently with a fine paintbrush onto six test leaves and two positive control leaves. In the control set the genotypes were mock inoculated by brushing leaves without mildew spores. Inoculated and mock-inoculated plant clones were placed in two separate growth chambers (Panasonic MLR-352) at 20 ± 2°C (day) and 16 ± 2°C (night) with 16:8 light-darkness (L:D) photoperiod, and randomly organized to minimize potential variation in microclimatic conditions. Two inoculated or mock-inoculated leaves were collected from every plant at 24, 48, and 72 h post inoculation (hpi), snap frozen in liquid nitrogen, and stored in glassine bags in -80°C until RNA extraction. Positive control leaves were screened 14 days post inoculation to confirm the plant phenotype as resistant or susceptible. Viability of spores used in the experiment was confirmed by inoculating detached leaves of a susceptible genotype.

### RNA Extraction

Altogether 0.2 g of frozen leaf material was ground in lysing buffer (2% CTAB, 2% PVP K-30, 100 mM *Tris*–HCl pH 8.0, 2 M NaCl, 25 mM EDTA), with β-MeOH (200 μl/10 ml) added in prior to use (Chang et al., 1993). Thoroughly vortexed solution was extracted twice with equal volume of acid phenol-chloroform-isoamyl-OH (ph 4.5). Prior to precipitation, 160 μL of 10 M LiCl was added and samples were kept on ice overnight, followed by 30 min centrifugation (10,000 rpm) in +4°C. Pellets were dissolved in 500 μL of 65°C SSTE (1 M NaCl, 0.5% SDS, 10 mm *Tris*–HCl pH 8.0, 1 mM EDTA) and RNA was extracted twice with chloroform-Isoamyl alcohol (24:1). After EtOH precipitation and 70% wash, the pellets were dissolved in 40 μL H_2_O and RNA quantity and quality were checked using NanoDrop (Thermo Fischer Scientific). Potential contamination of genomic DNA was removed using DNase I (Thermo Fischer Scientific) and samples were then reverse-transcribed to cDNA using iScript^TM^ cDNA Synthesis Kit (Bio-Rad) according to the manufacturer’s instructions.

### Quantitative Real-Time PCR

Three inoculated and mock-inoculated clones of two plant genotypes (resistant 193_2.1 and susceptible 1553_5.1) were sampled at three time points (24, 48, and 72 h post inoculation), resulting in 12 samples. Primers were designed with Primer3 (Rozen and Skaletsky, 1999) based on previously *in situ* sequenced transcriptome of Plantago (unpublished data) and known disease-induced genes in Arabidopsis. We tested seven putative disease-induced genes (Supplementary File 1). Amplification efficiencies (E) of the primer pairs were determined with five dilutions (1:1, 1:4, 1:24, 1:124, and 1:624) of template cDNA, where *E* = 10^–1^/slope. Three technical replicates, one water control and a plate control sample were included in a 384-well plate with 10 μL volume, using C1000^TM^ Thermal Cycler (Bio-Rad). All samples were tested for genomic DNA contamination with-RT controls prior to qPCR. Each reaction had 1 μL of the 1:4 diluted cDNA, 5 μL of SYBR^®^ Green containing master mix (iQ^TM^ SYBR^®^ Green Supermix for qPCR; Bio-Rad), 3 μL of nuclease-free water and 0.5 μL (10 μm) of each primer. The cycle conditions were one cycle at 95°C for 3 min, 40 cycles at 95°C for 10 s, 60°C 30 s, and ending with melting curve analysis. From the candidate set, Elongation factor_CL4, GADPH_28221 and Actin_34737 displayed a stable expression across the samples with geNorm and were selected as reference genes for normalization (Supplementary File 1). Relative expression (CNRQ) and normalization was calculated in qBase + 3.2.

### RNA Sequencing

Illumina paired-end sequencing (NextSeq 500) was carried out in the Institute of Biotechnology of the University of Helsinki with 78-base forward reads and 74-base reverse reads, with library insert size of 200 bases. Trimming and removal of low quality reads was carried out using Trimmomatic (Bolger et al., 2014), resulting in an average library size of 14.6 million reads.

### Transcriptome Assembly and Annotation

We combined all libraries for a pooled *de novo* assembly using Trinity (Grabherr et al., 2011), SOAPdenovo-*Trans* (*k*-mer sizes 39 and 41) (Xie et al., 2014) and Oases (*k*-mer sizes 39, 43, and 47) (Schulz et al., 2012). The combined assemblies contained 1,315,458 transcripts (177,948 from Trinity, 607,212 from SOAPdenovo, and 530,298 from Oases). The contigs were filtered with EvidentialGene (Gilbert, 2013). EvidentialGene pipeline removes redundant transcripts, transcripts with more than 98.5% similarity and internal ORFs, producing two sets of highly accurate transcripts referred to as okayset and okayalt set. The okayset are the most reliable set of transcripts from the original data while the okayalt set contains a set of alternative transcripts with many overlapping transcripts and some valid alternative transcripts. To increase the amount of alternative transcripts in our study the okayset and okayalt outputs were combined and clustered using RapClust (Srivastava et al., 2016). This procedure assigns transcripts with high sequence similarity to the same cluster. A representative transcript for each cluster was obtained using Lace (Davidson et al., 2017) which merges all the sequences in the same cluster to obtain a representative sequence. To remove contamination, the resulting contigs were queried against NCBI non-redundant protein database (Pruitt et al., 2007) using BLAST and transcripts with a best hit in plant kingdom were retained. The transcripts mapping to ribosomal genes and having ambiguous sites (Ns) were removed. Minimum read coverage of three was used for all the assemblies. A similar process was carried out for individual genotype transcriptome assemblies for comparison purposes.

For functional annotation of the transcripts, BLASTp (Camacho et al., 2009) with default cut-offs was used to find the best match among Arabidopsis representative set of proteins (Berardini et al., 2015), available at TAIR server(ftp://ftp.arabidopsis.org/home/tair/Proteins/TAIR10_protein_lists/). The functional annotation and gene ontology (GO) category assignment of the best Arabidopsis hit (from ftp://ftp.arabidopsis.org/home/tair/Ontologies/Gene_Ontology/) was then transferred to the Plantago query transcript.

### Differential Gene Expression Analysis and GO Enrichment

All libraries (30 in total) were mapped to the transcriptome assembly using kallisto (Bray et al., 2016) with 100 bootstrap replicates. The averages of bootstrap replicates of raw count values were used as counts. The count tables were imported to R by tximport package (Soneson et al., 2015). The validity of genotype-specific transcriptomes in the final assembly was assessed by mapping the raw sequencing reads to the transcriptome and identifying the proportion of transcripts expressed in a certain genotype. We required at least one genotype-specific read mapped to a transcript in at least two biological replicates. We conducted the analysis in R and using the count table from tximport. The overlaps of the common versus genotype-specific transcripts was visualized with a Venn diagram using VENN package in R.

Principal Component Analysis was carried out using DESeq2 and visualized with rgl package (Adler et al., 2017) for 3D plot and ggplot2 (Wickham, 2016) for 2D plots. DESeq2 package (Love et al., 2014) was used for differential expression analysis at genotype and phenotype levels (Res, Sus and Res_vs_Sus), with adjusted *p*-value of 0.1 as a threshold for significant differential expression, as recommended by DESeq2. To maximize the number of true positive transcripts, no fold change cut-off was used.

Gene ontology enrichments were analyzed using piano R package (Varemo et al., 2013). For threshold-based GO enrichments, GOAtools was used (Klopfenstein et al., 2018). To focus on signaling responses, the “responses” and “signaling” sub-trees of the Biological Process category were selected. The GO enrichments were plotted using gene set mean fold changes from the piano package.

### Redundancy Analysis

Vegan package (Oksanen et al., 2018) was used for redundancy analysis (RDA) and permutation test (permtest) with 10,000 permutations to test significance. The genes significantly contributing to the RDA loadings were identified using cut-off of three standard deviations (corresponding to two-tailed *p*-value = 0.0027 in *Z*-test). The overlap among gene sets was analyzed using venn package in R (Dusa, 2018). The GO enrichment of RDA loadings was carried out with piano (Varemo et al., 2013) using RDA loadings as gene level statistics.

### Prediction of Candidate NLRs

The candidate NLRs were predicted using NLR-Parser (Steuernagel et al., 2015). The highest scoring domain found per reading frame per transcript was picked and screened manually. The transcripts were filtered out if the ORF was too short, if the start and stop codons were missing, or if BLAST queries did not return hits to NCBI non-redundant database. To account for partial or miss-assemblies, we performed an online search for NB-ARC domain in NCBI Web CD Search Tool (Lu et al., 2020), and selected the transcripts with a complete NB-ARC domain. Both protein and nucleotide sequences of these domains were extracted for subsequent analyses.

To identify the NB-ARC domains contributing to the separation of the phenotypes, the full NLR transcripts were replaced with their NB-ARC domains and the reads were remapped using kallisto. RDA analysis on NB-ARC domains was carried out with vegan R package, using significance cut-off of one standard deviation. Differential expression was calculated with TPM-normalized values (Wilcoxon test with Benjamini-Hochberg correction).

For evolutionary analysis, *Antirrhinum majus* L. (snapdragon) was used as outgroup, since it is the most recently diverged plant with published chromosome-level genome assembly (Li M. et al., 2019). NLR transcript prediction with complete NB-ARC domains was carried out using the protocol described above. Multiple sequence alignment of the NB-ARC domains was carried out using MAFFT, and the phylogenetic tree was estimated using RAxML v8 (Stamatakis, 2014) with PROTGAMMAAUTO and 100 bootstrap trees. The tree was cut with ClusterPicker (Ragonnet-Cronin et al., 2013) with 90% initial threshold and main support threshold for clusters and genetic distance of 0.2 with gap option into clusters. To get an ancestral state, the most common snapdragon protein hit among BLAST queries with the cluster sequences was added to the cluster. The gene tree was visualized with ggtree R package (Yu et al., 2017). The putative Arabidopsis homeolog was selected with BLAST query of the full transcript against the TAIR proteome.

### Neutrality Test (dN/dS and H Statistic)

Multiple sequence alignment for each cluster was carried out using MAFFT and gene tree was estimated with FastTree (as suggested in PAML manual), followed by reverse-transcription of the aligned sequences. Gene and per-base level dN/dS ratios (non-synonymous versus synonymous mutations) were calculated using PAML (Yang, 2007), with dN/dS > 1 indicating positive selection.

For Fay and Wu’s (2000) H statistic, the reads were aligned to the transcriptome containing NB-ARC domains with BWA (Li and Durbin, 2009) and using ANGSD (Korneliussen et al., 2014) to calculate H statistic within a sliding window of three nucleotides. *H* values <-3 signified positive and >1 purifying selection, respectively. Overall nucleotide diversity (π) and Watterson theta (θ) were obtained as the mean over transcripts and then over the transcriptome.

## Results

**FIGURE 1:**
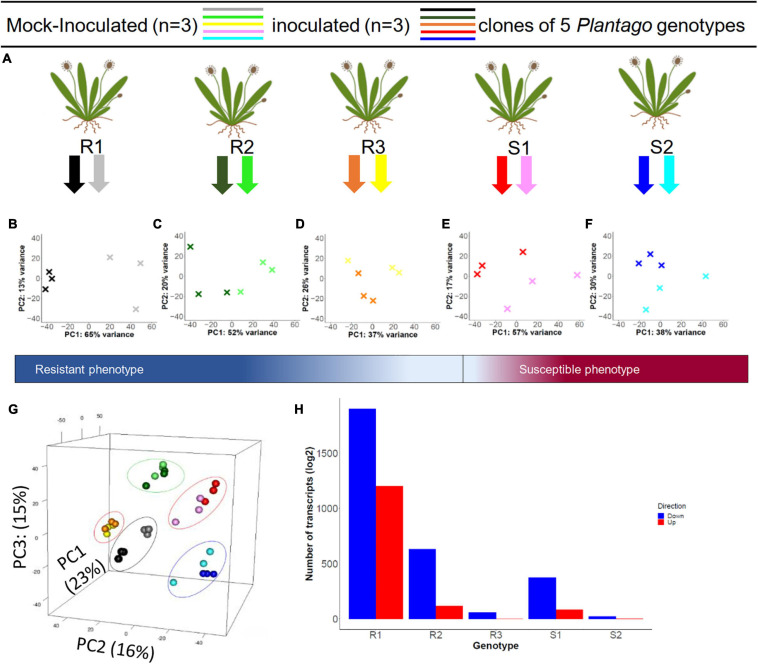
Experimental design of the inoculation experiment and principal component analysis of the resulting differential gene expression in five *Plantago lanceolata* genotypes. **(A)** For each of five *P. lanceolata* genotypes, three clones were inoculated with a strain of *Podosphaera plantaginis* and three clones were mock-inoculated as control. RNA was extracted at 72 h post inoculation, and the phenotypes (resistant or susceptible) were scored at day 14 post inoculation. **(B–F)** The effect of the inoculation treatment within each genotype is illustrated with Principal Component Analysis (PCA) where the first two axes (PC1 and PC2) explain between 37–65 and 13–30% of the variation. All plots show a clear treatment effect. **(G)** When analyzed together, the genotypes separate by their gene expression along PC1, PC2, and PC3 axes, which explain 23, 16, and 15% of the variation, respectively. **(H)** Within genotypes, R1–3 and S1–2 showed varying numbers of significantly up (red) and down (blue) regulated genes in response to the inoculation treatment.

A schematic overview of the experiment design is shown in Figure 1A. In order to choose the most informative time point for analysis we studied the expression of a selected set of marker genes using qPCR, taking into consideration that the development time of *P. plantaginis* is relatively slow compared to agricultural powdery mildews: It typically takes between three to seven days for mycelia to become visible on the leaf surface, and the conidial spores appear on average seven days post inoculation (Green et al., 2002; Laine, 2007). We detected powdery mildew spores growing on the inoculated, susceptible plant leaves on day 14 post inoculation, whereas none of the mock-inoculated or inoculated resistant plant clones showed visible disease symptoms. Several studies of gene expression induced by powdery mildew in host plants have found the highest number of differentially expressed genes in later time points (Li et al., 2016, 2019; Polonio et al., 2019). Accordingly, transcript levels of the marker genes varied considerably in the susceptible plants and showed elevated levels only at time point 72 h post inoculation (hpi; Supplementary Figure 1) and therefore this time point was selected for RNA sequencing.

### Transcriptome Assembly and Expression Analysis

The pooled assembly contained altogether 1,315,458 transcript models, which were clustered into 86,648 transcripts using EvidentialGene. The resulting transcriptome was of high quality, since 87% of the universal single-copy genes (BUSCO; Seppey et al., 2019) were present (including complete and fragmented genes), but the high proportion of duplicated gene models (46.3%) suggested the presence of many splice variants and allelic variants. Subsequent clustering and merging (see M&M) resulted in 24,332 high quality non-redundant transcripts with an average length of 1,858 bases. The procedure reduced the Busco score to 77.5%, but clearly removed the allelic variants, as only 2% of gene models remained duplicated (Supplementary Table 1). The filtered gene models had mostly low expression counts and therefore were of low biological significance to the experiment; this was clearly visible from the mapping rates, as on average 83 and 75% of the transcriptome data mapped to the raw and filtered *de novo* assemblies, respectively (Supplementary Table 2). The final assembly was representative of all genotypes since about 92% of the transcripts were expressed across all genotypes (Table 1). The Venn diagram of the expression profiles shows that less than 1% of the transcripts were expressed in only one genotype (Supplementary Figure 2).

**TABLE 1:** The percentage of expressed transcripts per genotype.

Group	S1		S2		R1		R2		R3	
Inoculated	22,628	93%	22,418	92%	22,680	93%	22,548	92%	22,312	91.6%
Control	22,567	92.7%	22,457	92%	22,431	92%	22,484	92%	22,319	91.7%

*The percentage of transcripts that have reads mapped to them in at least two biological replicates per genotype in control and experiment. About 92% of the transcripts are expressed across all genotypes. This is an indication of the transcriptome being a good representative of all genotypes.*

We detected high nucleotide diversity (π = 0.068) and Watterson theta (θ = 0.077) for the transcriptome. Principal Component Analysis (PCA) of TPM normalized gene expression data showed a clear grouping by genotype (Figure 1G) along the first three PCs. These first three PCs explained altogether 53% of the total variation, illustrating that genotype is the main contributor to the variation between samples. The inoculation treatment had a smaller but marked effect, as was clearly demonstrated in genotype-specific PCA plots (Figures 1B–F). For example, in resistant R1 and R2 genotypes the variation explained by PC1 was 65 and 52%, respectively, and clearly separated the inoculated and control plants (Figures 1B,C).

**FIGURE 2:**
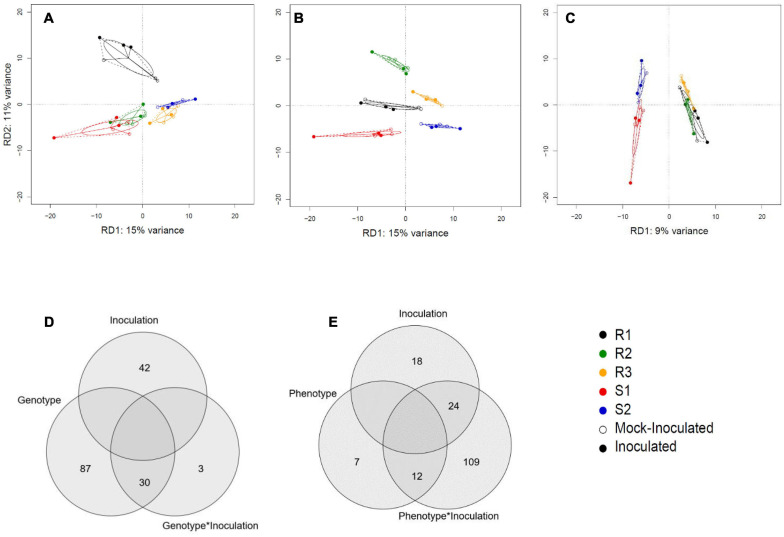
Redundancy analysis (RDA) of RNASeq data. **(A)** Discriminative axes RD1 and RD2 explain 15 and 11% of the genotype variation, respectively. **(B)** The genotypes are best separated when RD1 is plotted against RD3 (explaining 9% of the variation). **(C)** The susceptible vs resistant phenotype split explains 9% of the variation. Results are displayed against principal component 1 of the remaining variation (PC1) as there is only one RD axis for phenotype difference. **(D)** A Venn diagram illustrating the number of genes that contribute significantly to variation in genotype, inoculation, and genotype × inoculation interactions. **(E)** A Venn diagram shows the number of genes that contribute significantly to variation in phenotype, inoculation, and phenotype × inoculation interaction.

We next characterized the overall effect sizes and their statistical significance using Redundancy Analysis (RDA; multivariate linear regression). The genotype and phenotype effects were highly significant (*P* = 0.001 and *P* = 0.004), explaining 35 and 9% of the overall variation in gene expression (Table 2). The effect of inoculation alone was not significant (*P* = 0.238), but the combined effects of genotype-by-inoculation and phenotype-by-inoculation were (*P* = 0.001 and *P* = 0.048, respectively), suggesting genotype-specific response profiles. Accordingly, the RDA plots displayed clear separation when using genotype and phenotype as a covariate but not with inoculation treatment alone (Figures 2A–C). Venn diagrams of the genes contributing to the separation in the RDA demonstrate that while 87 genes contribute significantly to separation according to genotype, only seven genes contribute directly to the phenotypic variation and 109 genes to the joint effect of phenotype-by-inoculation (Figures 2D,E).

**TABLE 2:** A summary of the Redundancy Analysis (RDA) results.

Source	*P*-value				Percentage of variation (%)				Total percentage of variation
	RDA1	RDA2	RDA3	RDA4	RDA1	RDA2	RDA3	RDA4	(%)
Genotype	0.001	0.001	0.001	0.002	15	11	9		35
Phenotype	0.004	NA	NA	NA	9	NA	NA	NA	9
Inoculation	0.238	NA	NA	NA	4	NA	NA	NA	4
Genotype × Inoculation	0.001	0.001	0.005	0.021	17	12	9	8	46
Phenotype × Inoculation	0.048	0.413	0.823	NA	9	4	2	NA	14

*P-values of RDA axes for Genotype, Phenotype, and Inoculation treatment effects and their interactions, and the percent of variation explained by each of the axes as well as the total variance explained.*

### Differential Gene Expression Analysis

Similarly, the differential expression analysis between mildew inoculated and mock-inoculated plants showed marked differences between the genotypes and their responses to the inoculation. The R1 genotype had the highest number of differentially expressed (DE) transcripts (3803), from which about 2,000 had absolute log_2_ fold change greater than one. On the other hand, the S2 genotype had the lowest number of DE transcripts, 43, with only 20 having absolute log_2_ fold change greater than one (Figure 1H, Table 3 and Supplementary Table 3).

**TABLE 3:** A summary of differentially expressed (DE) genes.

Sample	DE transcripts	|logFC| > 1	DE NLRs	DE NLRs |logFC| > 1
Sus1	791	166	10	3
Sus2	43	20	0	0
Res1	3,803	1,987	70	30
Res2	1,180	412	17	4
Res3	111	7	1	0
Sus	3,787	1,212	104	54
Res	1,108	143	13	0
Res_vs_Sus	3,912	1,325	107	45

*The table shows the number of differentially expressed transcripts (DE transcripts), the number of transcripts with large changes (|log FC| > 1), and the numbers of differentially expressed NLR transcripts (DE NLRs) and NLRs with large expression changes (DE NLRs |log FC| > 1) in inoculation vs. mock comparison. The numbers are reported for five genotypes separately, for the combined effect of susceptible (Sus) and resistant (Res) phenotypes, as well as their comparison (Res_vs_Sus).*

With default BLAST cut-offs and searches at protein level all transcripts obtained a hit to Arabidopsis proteins. The evolutionary distance of *Plantago* and Arabidopsis is relatively large (approx. 120 million years), but we nevertheless expect the active protein domains to be well-conserved in plant kingdom; in fact there exists evidence of conserved protein structures even from far longer time spans (Vaattovaara et al., 2019). Since the GO categories are based on protein domains, this should give a good estimate of the putative GO assignments for *Plantago*.

**FIGURE 3:**
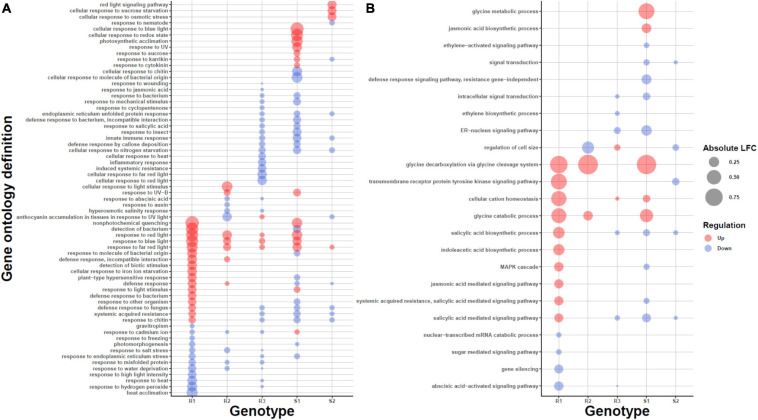
Gene Ontology (GO) enrichments of the Differentially Expressed (DE) transcripts in the different genotypes R1–3, S1–2. Statistically significantly enriched GO categories among the DE transcripts for categories related to **(A)** biotic and abiotic responses and **(B)** signaling. The size of the bubble is proportional to the mean fold change of the genes assigned to the GO category and the color describes the direction, upregulation (red) or downregulation (blue).

To obtain a higher-level characterization we next looked for common enriched pathways among the plants using GO enrichment analysis. The differences among expression profiles was also visible in the level of pathways (Figure 3; Supplementary Figure 2, and Supplementary Table 4). We first compared susceptible and resistant genotypes by searching for GOs with decreased average expression levels in susceptible phenotypes and elevated levels in resistant phenotypes. In resistant phenotypes, genes encoding photosynthesis-related proteins (e.g., Photosystem II antenna complex and chloroplast photosystem I/II) and NAD(P)H dehydrogenase complex had increased transcript levels (Supplementary Figure 2); genes assigned to photosynthesis functions showed elevated transcript levels in susceptible phenotypes as well but not to the same extent. On the other hand, in susceptible phenotypes the GO category with most decreased expression levels was induction of programmed cell death (GO:0012502) (Supplementary Figure 2), suggesting that as a biotrophic pathogen, *P. plantaginis* may be downregulating the programmed cell death to keep the host cells alive. However, also the expression levels of resistant phenotypes were reduced in this category, possibly due to successful manipulation by the pathogen, and therefore the comparison between susceptible versus resistant did not identify this process as significantly different between phenotypes (*P* = 0.0559).

In addition to the shared responses, the genotypes showed individual enrichment of various disease resistance pathways (Supplementary Figure 2). In susceptible genotype 1 (S1), the processes with most decreased average expression levels were “tripeptide transporter activity” (GO:0042937), “tripeptide transport” (GO:0042939) and “delta12−fatty acid dehydrogenase activity” (GO:0016720), whereas S2 demonstrated decrease in “oxazole or thiazole biosynthetic process” (GO: 0018131) and “low−affinity nitrate transport” (GO:0080054 and GO:0080055). Fatty acids play a direct role in modulating the plant defense response to pathogens (Kachroo and Kachroo, 2009), and thiazole or thiamine has been shown to play a crucial role in activation of the defense responses, callose/lignin deposition and stomatal closure (Zhou et al., 2013). Tripeptide transport includes also nitrate transporters. Interestingly, powdery mildew causative agent *Erysiphe necator* elevates the expression levels of nitrate transporters in grapevine and Arabidopsis (Pike et al., 2014), possibly to acquire nutrients from the host. In addition to decreased levels of the GO categories related to nitrate transport in both S1 and S2, we identified homolog of Arabidopsis nitrate transporter (AtNRT1.5) to be upregulated after inoculation in susceptible vs resistant comparison. In Arabidopsis, the protein is responsible for nitrate transport from roots to shoots, and in this context suggests toward manipulation of host nutrient distribution by the pathogen. In general, tripeptide transport also plays an important role for defense against biotic and abiotic stress (Karim et al., 2007), suggesting a reason for the decreased expression of the tripeptide transporters as a whole in the susceptible phenotypes. Nitrogen, nitrates and their transport to different tissues in the plant during the pathogen infection could play a critical role in the plant defense (Mur et al., 2017).

In resistant phenotypes, the glucosyltransferase (GO:0050284) upregulation in R1 is a possible sign of early preparation for pathogen response (Le Roy et al., 2016), and in R2 genotype, the activation of NADH dehydrogenase complex assembly (GO:0010258) has been shown to be involved in defense signaling (Wallstrom et al., 2014).

### Gene Ontology Enrichment Based on Redundancy Analysis

Activation of plant signaling pathways involves coordinated expression changes of many genes in the pathway and therefore is multivariate in nature. Hence, we applied RDA to look for GO categories differentially activated between the phenotypes or the treatments by calculating the average RDA loadings (Supplementary Table 5) of the genes in the categories and tested for their statistical significance. Genes contributing to the separation between inoculation and control were enriched for ABA and cytokine signaling, primary metabolism and chloroplast activity (Supplementary Figure 3). ABA induces resistance to powdery mildew in barley (Wiese et al., 2004), and repression of ABA biosynthesis as well as genes regulated by ABA, such as cold/dehydration/salinity responsive genes, are associated with mildew resistance in non-host plants in general (Jensen et al., 2008). Cytokinin suppresses programmed cell death and plays a role in the synthesis and maintenance of chlorophyll (Walters and McRoberts, 2006) (Supplementary Figure 3). Additionally, cytokinin levels regulate cell division together with auxin. Interestingly, in Arabidopsis, *Golovinomyces orontii* inoculation induced cell cycle related genes and endoreduplication, possibly due to increased metabolic demands of the pathogen (Chandran et al., 2010). On the other hand, Choi et al. (2011) have shown that plant based cytokinins systematically induce plant resistance against pathogens by cytokinin and salicylic acid signaling.

Genes associated with the differences between the phenotypes showed GO enrichments for kinase activity, carbohydrate metabolism, plant cell wall organization, photosystem II and response to cold GO categories (Supplementary Figure 3), whereas the genes contributing to the differences between genotypes were enriched for tryptophan metabolism, plant cell wall and chloroplast (Supplementary Figure 3). In Arabidopsis (Chandran et al., 2010), the expression of cold/drought responsive genes were decreased together with ABA biosynthesis after inoculation with *G. orontii*. Together with the observed induction of ABA during inoculation, this suggests that the phenotypes may differ in how strongly ABA activates its targets such as cold responsive genes.

**FIGURE 4:**
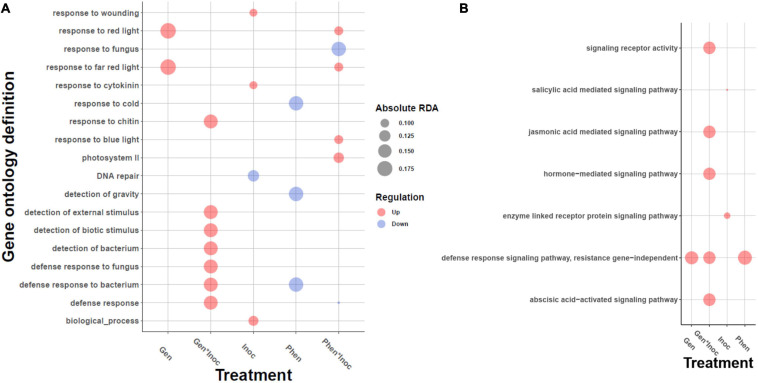
Gene ontology enrichments of the genes contributing to the redundancy analysis (RDA) axes for treatment, genotype and phenotype levels. Enrichment test was carried out using gene loadings along RDA axes, see “Materials and Methods” section. The bubble plot depicts **(A)** biotic and abiotic responses, and **(B)** enrichment limited to signaling hierarchies. The size of the bubble is proportional to the mean fold change of the genes assigned to the GO category and the color describes the direction, upregulation (red) or downregulation (blue).

Different responses to infection are visible in the genotype-by-inoculation effect. Overall, the enriched GOs show a clear activation of defense responses in general, and defense responses to fungi in particular (e.g., regulation of immune response, regulation of defense response; Supplementary Figure 3), illustrating that the genes in these processes differ in their transcription levels between genotypes. The GO category with highest positive average of RDA loadings (and therefore, high contribution to separation) is aldose 1−epimerase activity (GO:0004034) which may be activated because of the mechanical damage inflicted by the pathogen and results in methanol emission and priming of the non-infected leaves (Sheshukova et al., 2017). Next, hydrogen peroxide metabolic process and salicylic acid mediated signaling pathway are both well-established pathogen-induced defense mechanisms (Kuniak and Urbanek, 2000; Hua, 2009; Niu and Liao, 2016; Sheshukova et al., 2017), further demonstrating the activation of the defense processes due to the pathogen infection. The GO category with most negative average RDA loadings is RNA splicing, via endonucleolytic cleavage and ligation (GO:0000394). It is becoming increasingly clear that plants use alternative RNA splicing extensively as a means to respond to their environment and defend against pathogens (Staiger et al., 2013; Shang et al., 2017). Within the signaling-specific GOs (Figure 4) the genotype-by-experiment effect showed the increased transcript levels of jasmonic acid (JA) and abscisic acid signaling [as expected, (Yang et al., 2019)], again in a genotype-specific manner. Further inspection of putative orthologs of marker genes for different hormonal signaling pathways showed increased transcript levels of auxin biosynthesis and signaling, as well as differences in the increased transcript levels of JA signaling and NLR signaling through EDS1 ortholog (Supplementary Table 6).

The most significant contributor to phenotype-by-experiment is photosystem II activity (Supplementary Figure 3), as several GO terms from this category showed significant enrichments. The GO category with highest average RDA loadings for phenotype-by-inoculation is oligopeptide transmembrane transporter activity (GO:0035673). The perception and transduction of fungal oligopeptides will trigger multiple defense responses (Nürnberger et al., 1994; Hahlbrock et al., 1995). Multitude of photosynthetic processes were also enriched; their role in defense was discussed above. The categories with most negative average loadings were response to fungus, and cytokinin biosynthetic process (GO:0009691).

### NLR Transcripts

When we assemble genotypes individually, we obtained 368, 284, 239, 256, and 191 NLR transcripts for genotypes S1, S2, R1, R2, and R3, respectively. The combined assembly on the other hand contains 543 NLR candidates that is almost twice as much as each of the individuals assembled separately. This is an indication that our combined transcriptome assembly should be a good representative of the NLR repertoire of the individuals that we are studying. To look for the variation in the plant defense arsenal we carried out an in-depth study of the resistance NLR genes induced in the experiment. The highly repetitive nature of the LRR domain is known to be problematic in *de novo* assembly from short-read RNA sequencing data, and therefore we focused the analysis on the conserved NB-ARC domains. Out of the 543 candidate NLR transcripts in the transcriptome, 210 had a complete NB-ARC domain.

**FIGURE 5:**
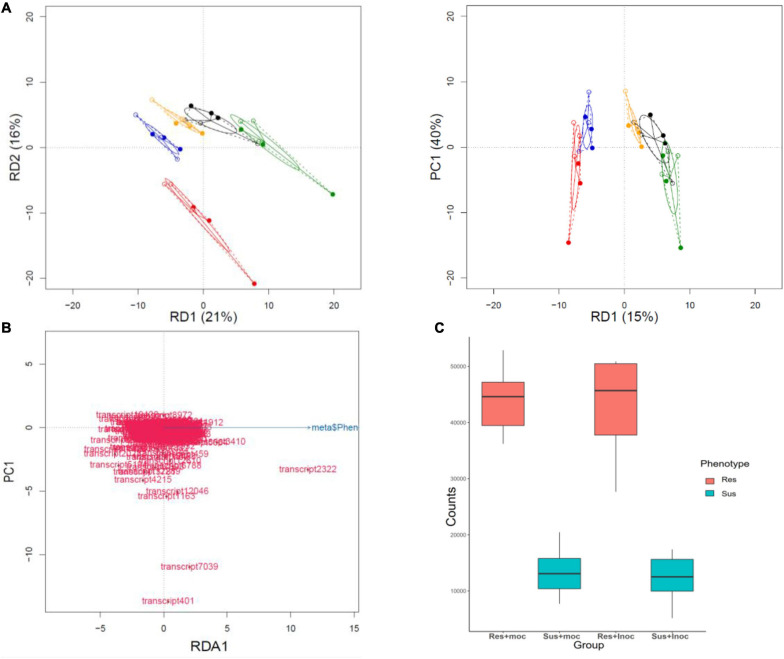
Redundancy analysis of NB-ARC domains. **(A)** (Genotype) RDA analysis of the genotype effects of NB-ARC domain expression data explains 55% of the variation (four axis only two are depicted here). (Phenotype) the same analysis shows that phenotype explains 15% of the variation that separates the resistant and susceptible phenotypes. **(B)** NB-ARC sequences contributing to the separation of the phenotypes (Resistant vs Susceptible); transcript2322 has the highest contribution to the separation. A full-length BLAST query of this transcript against the TAIR database returns RPP13 as the best hit (Bit score 216 and E-value 4e-60). **(C)** Comparison of the expression values (TPM) of transcript2322 reveals that even though this transcript is not differentially expressed due to the experiment, it has significantly higher (*P*-value = 0.0003996) base expression in resistant phenotypes before and after the experiment. This could be due to maternal effects or different regulation of the gene in resistant phenotypes.

The inoculation did not have a significant effect on expression levels in RDA analysis of NB-ARC domains (*p* = 0.13), but the genotype and phenotype both contributed significantly (*p* = 9.999e-05 and *P* = 4e-04), explaining 55 and 15% of the variation, respectively (Figure 5). Based on RDA loadings, the NLR transcript with highest contribution to resistance phenotype was transcript2322, a putative homolog of Arabidopsis AtRPP13 gene. RPP13 has the highest amount of amino acid diversity among Arabidopsis proteins (Rose et al., 2004) and is involved in defense against *Peronospora parasitica* (Rose et al., 2004; Hall et al., 2009), an oomycete causing downy mildew in Brassicaceae. The transcript2322 was not significantly differentially expressed due to inoculation, but it had significantly higher base expression level (*p*-value = 0.0003996) in resistant phenotypes (Figure 5).

**FIGURE 6:**
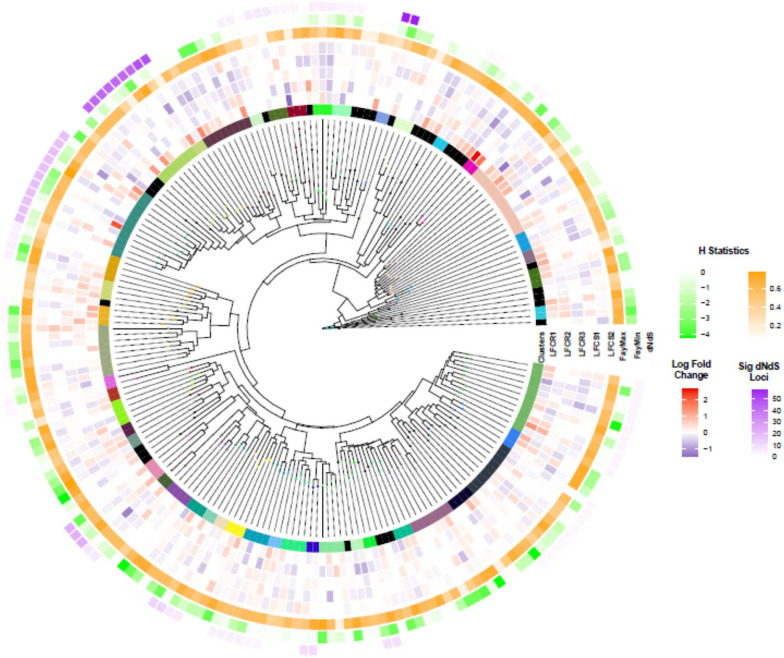
Nucleotide-binding–leucine-rich repeat (NLR) transcripts: clustering, differential expression, dN/dS, and Fay H statistic. The phylogenetic tree of NB-ARC domain of NLR transcripts. The different colors in the innermost circle illustrate cluster assignments (track denoted as “Clusters”). The next tracks show the log_2_ fold change (LF) of the transcript in each of the five genotypes (LFCR1-3, LFCS1-2; R, resistant; S, susceptible). FayMax and FayMin show minimum and maximum value of H statistic. Finally, dN/dS illustrates the number of loci with significant dN/dS value in each cluster, the color intensity is proportional to the number of loci.

Clustering of the NB-ARC domain tree resulted in 47 clusters containing 179 sequences and 31 singletons (Figure 6), Cluster 4 being the largest with 12 sequences. BLAST query against Arabidopsis proteome for the longest transcript in this cluster returned a hit to AT3G14460, a leucine rich repeat protein that also contains an adenylate cyclase catalytic core motif. This gene is involved in adenylyl cyclase activity and signaling and its knockouts in Arabidopsis have compromised immune responses to the biotrophic fungus *Golovinomyces orontii* (Bianchet et al., 2019).

### Neutrality Test (dN/dS and H Statistic)

To look for NLR clusters under positive selection, we analyzed dN/dS, the ratio between non-synonymous (amino acid changing) to synonymous mutations (Figure 6). None of the clusters appeared to be under positive selection. However, site-wise analysis of dN/dS revealed that 25 of the clusters contained a varying number of one to 58 amino acid positions under positive selection, based on Bayes Empirical Bayes (BEB) analysis (*P* > 95%). Cluster 14 with the highest number of loci under selection returned Arabidopsis NLR protein AT1G50180 (CAR1) as the best BLAST hit, an immune receptor which recognizes the conserved effectors AvrE and HopAA1 (Laflamme et al., 2020).

In order to investigate potential selection pressure by a complementary method, considering the shortcomings of within population dN/dS analysis (Kryazhimskiy and Plotkin, 2008), we also calculated Fay and Wu’s H statistics for the NB-ARC domains. A positive value of H indicates balancing or purifying selection, whereas high negative values indicate positive selection in the form of selective sweeps, or drift, for example from population bottlenecks. We identified 27 NLR transcripts with H values less than -3 (Figure 6; Supplementary Figure 4, and Supplementary Table 7). This set included one gene from the cluster with the highest number of loci under selection based on dN/dS analysis, as well as the transcript2322 having significantly elevated expression levels in the resistant vs susceptible comparison. BLAST query of the NB-ARC domains under selection against TAIR database resulted in 16 hits to RPP13 and three hits to CAR1 (Supplementary Table 7).

## Discussion

Natural host populations have been shown to support considerable diversity in resistance (Salvaudon et al., 2008; Laine et al., 2011), and theory predicts that this variation is maintained by pathogen-imposed selection. With recent advances uncovering the molecular underpinnings of resistance, it is becoming increasingly feasible to study this diversity also in non-model systems. Here, we established a high-quality *de novo* transcriptome assembly of *P. lanceolata* to investigate the gene expression and processes activated in five plant genotypes in response to inoculation of the same pathogen strain. All five plant genotypes showed unique gene expression patterns, as was demonstrated already by the PCA. In further analysis, the inoculation explained only 4% of the total variation, while genotype-by-inoculation interactions contributed 46%. Surprisingly, split to susceptible vs. resistant genotypes explained only 9% of the variance, with considerable expression pattern differences between phenotypes. Overall, the plant genotypes differed by the number, fold change and the function of the transcripts differentially expressed in response to the pathogen although the effect of time point on gene expressions could not be ruled out. High variation in gene expression patterns among plant genotypes has also been discovered in other studies (Burghardt et al., 2017; Muller et al., 2019).

Diversity was evident already in signaling associated with pathogen response; all phenotypes showed the expected induction of JA, SA, and ABA signaling pathways, but to a highly varying manner, with JA and ABA significantly contributing to the genotype by experiment differences. This variation in the extent of activation of signaling pathways and resulting differences in cross-talk could be an important mechanism generating phenotypic resistance diversity. The diverse responses may be linked with the high genetic variation within the species, with Watterson θ = 0.068 and nucleotide diversity π = 0.077, meaning that on average, eight nucleotides out of 100 differ between any two individuals. The experimental take home message is that including multiple genotypes in experiments and avoiding pooling for RNA-Seq is essential to uncover variation relevant for phenotypic differentiation.

The pathways commonly induced by the pathogen included the induction of specific nitrate transport genes in susceptible phenotypes as well as elevated expression of photosynthesis-associated genes and related biological processes. This could contribute in defense against the pathogen, since photosynthesis in known to play an important role in plant defense against biotic stress (Gohre, 2015). Chlorosis is a hallmark sign of powdery mildew infection and biotrophic fungi are known to reduce photosynthetic rate and possibly damage chloroplast structure (Perez-Bueno et al., 2019), thus the upregulation could be either compensation, plant defense mechanism or induced by pathogen. Specifically, uroporphyrinogen decarboxylase activity (GO:0004853) was upregulated in resistant phenotypes (Supplementary Figure 2, Resistant). Involved in chlorophyll biosynthesis, it also points toward acting against the chlorosis induced by the pathogen (Mock et al., 1998). Powdery mildew fungi have a contracted carbohydrate metabolism, for example they are not able to degrade pectin, an essential component of plant cell walls (Liang et al., 2018), whereas the lipid metabolism is intact, suggesting that their main source of energy is from lipids. The elevated expression of specific nitrate transporters and chloroplast processes suggests elevated chlorophyll biosynthesis. Together with the chlorosis phenotype, this suggests that *P. plantaginis* may target the chloroplast lipids to obtain its nutrients. However, at this stage this is a hypothesis that is supported by previous studies on the genome structure of other powdery mildews and the observed GO enrichments in our data. More specific molecular work is needed to truly understand the photosynthetic response of *P. lanceolata* to *P. plantaginis*.

### Discovery of a Diverse Repertoire of NLRs in *P. lanceolata*

Nucleotide-binding–leucine-rich repeats play an important role in pathogen recognition, defense responses and signaling, as well as activation of hyper-sensitive response (Monteiro and Nishimura, 2018). Our transcriptome assembly contained 543 NLR isoforms, with 210 transcripts having a complete NB-ARC domain. A majority of these transcripts were expressed to some extent in all five plant genotypes. Presence-absence polymorphism in a subset of NLRs has been demonstrated across Arabidopsis accessions (MacQueen et al., 2019; Van de Weyer et al., 2019), and hence it could contribute to the slight differences in the numbers of NLRs detected in the genotypes.

The NLR transcripts with a complete NB-ARC domain divided into 47 clusters, with 12 transcripts in the largest cluster. NLR genes are among the fastest evolving gene families in plants, and we found considerable variation in the branch lengths among clusters, possibly indicating different evolutionary rates (Tucker et al., 2013). The NLRs often form tandemly arrayed gene clusters, which may be a critical contributor to their fast pace of structural and functional diversification (Michelmore and Meyers, 1998; Meyers, 2003). Frequent homologous recombination events and errors produced during the process, followed by diversifying selection, may generate the structural diversity needed to match high effector evolution rates in the pathogens (McDowell and Simon, 2006; Jacob et al., 2013). In particular, we found multiplication in the number of homologs of Arabidopsis RPP13 across several clusters (Supplementary Table 7). RPP13 is involved in defense against downy mildew (*Peronospora parasitica*) in Arabidopsis, as well as other defense processes and signaling (Bittner-Eddy et al., 2000; Rentel et al., 2008), and one of these paralogs showed differing expression patterns in resistant vs susceptible phenotypes. While none of the clusters had significant dN/dS values, several individual loci were found to be under selection in 25 of the clusters. The Arabidopsis homolog of the cluster 14, having the highest number of loci under selection, has been suggested to be involved in recognition of the conserved effectors AvrE and HopAA1 (Laflamme et al., 2020). The H statistic identified the same transcripts as the dN/dS analysis (18 transcripts), plus four other NLRs, including the specific homolog of RPP13 with high expression values in resistant phenotypes. Altogether, the analysis suggests this gene to be a good candidate for further studies.

Overall, the NLR transcripts were differentially expressed in response to the pathogen treatment and this response varied according to genotype. While we observed elevated NLR expression levels in response to the pathogen, this was not consistent across transcripts and genotypes, which is in line with recent studies on crop plants testing different genotypes (Sari et al., 2017, 2018; Cruz-Miralles et al., 2019). Plants have evolved mechanisms to stabilize their basal expression levels, and to reduce the fitness costs of an overexpressed immune response that could have more deleterious effects on plant fitness than the infection (Fei et al., 2013). This may explain the down-regulation of some of the NLR transcripts we observe in both susceptible and resistant phenotypes. We acknowledge that temporal analysis of expression patterns could yield further insight into the dynamics of the resistance response. Future studies are needed to determine how sensitive the detection of NLRs and their expression patterns are to the sampling time.

## Conclusion

Characterizing the architecture of resistance in natural host populations may yield unprecedented light on the potential of evolution to generate variation, and it can have broad and long-lasting impacts in our food production environments. Toward this end, we studied the transcriptional response of *P. lanceolata* to its obligate biotroph pathogen, *P. plantaginis*. Our analysis demonstrated that resistance emerges as a result of crosstalk between plant’s defense mechanisms and signaling pathways and although there are some similarities between resistant and susceptible phenotypes the differences manifest themselves in a genotype-dependent manner. We further studied the repertoire of candidate NLRs in *P. lanceolata* and found evidence of selection generating diversity in a subset of the identified NLRs, with one candidate NLR showing significantly elevated expression levels among resistant phenotypes and signs of positive selection. This study highlights the importance of investigating resistance responses across several genotypes as they may contribute to population-level resistance via different resistance strategies. This could have major implications for our understanding of the evolution of disease resistance in plants in the wild, and has the potential to guide improved crop resistance and food security.

## Data Availability Statement

The datasets presented in this study can be found in online repositories. The names of the repository/repositories and accession number(s) can be found below: https://www.ncbi.nlm.nih.gov/, Accession: PRJNA636383.

## Author Contributions

A-LL, JS, PS, and LH conceived the ideas. JS, PS, LH, MB, and A-LL designed the experiment. LH conducted the experimental work. PS, JS, and LH analyzed the data. PS, JS, and A-LL led the writing of the manuscript. All the authors contributed to the drafts and gave final approval for publication.

## Conflict of Interest

The authors declare that the research was conducted in the absence of any commercial or financial relationships that could be construed as a potential conflict of interest.
