# Supplementary material for: Genotype-Specific Expression and NLR Repertoire Contribute to Phenotypic Resistance Diversity in Plantago lanceolata
Source: Front Plant Sci. 2021 Jul 12;12:675760. doi: 10.3389/fpls.2021.675760 (PMC8311189; doi:10.3389/fpls.2021.675760)
Supplement: Supplementary file 1 [file Data_Sheet_1.zip › Supplementary_Figures.pptx]

## Slide 1
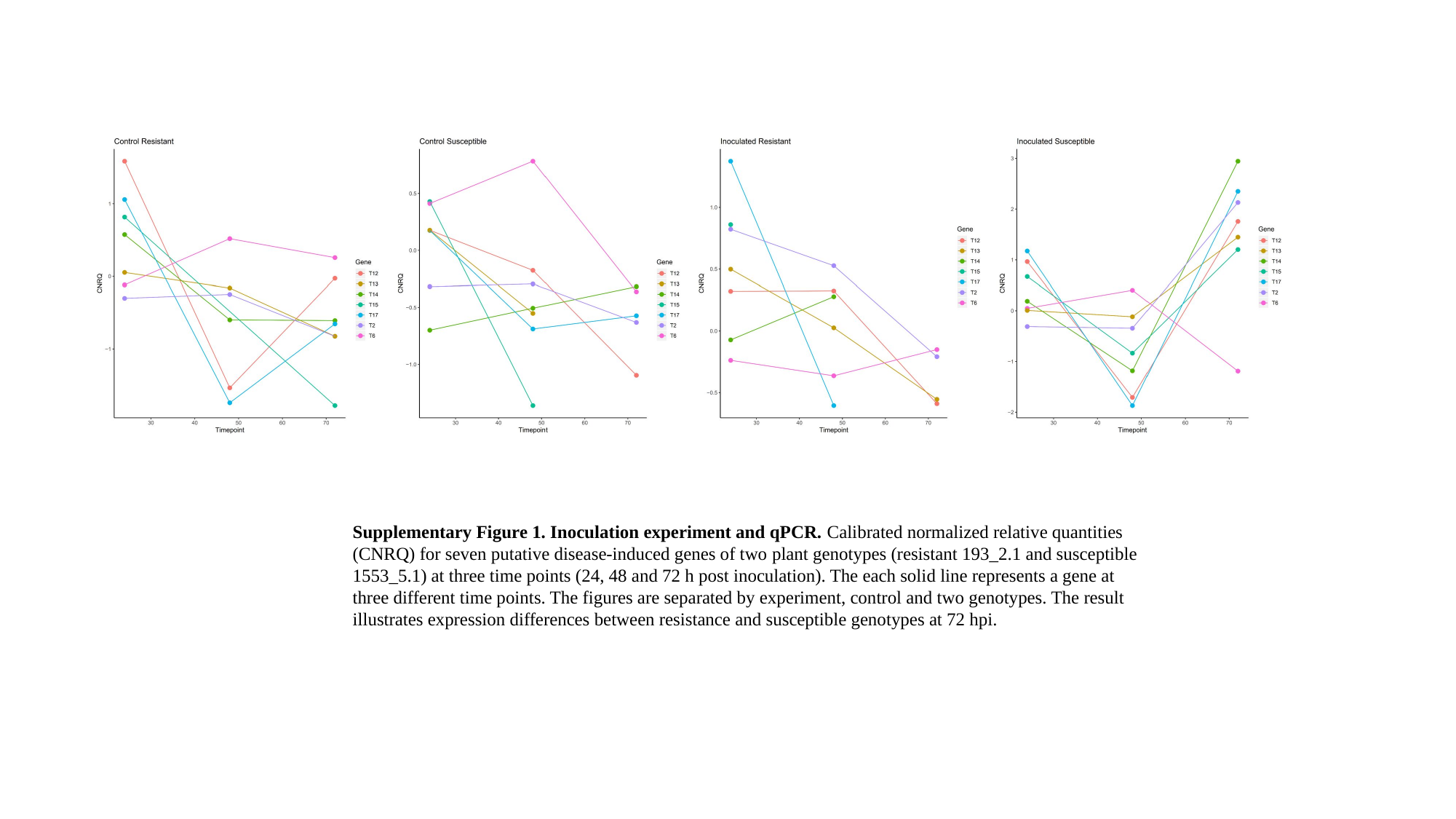

Supplementary Figure 1. Inoculation experiment and qPCR. Calibrated normalized relative quantities (CNRQ) for seven putative disease-induced genes of two plant genotypes (resistant 193_2.1 and susceptible 1553_5.1) at three time points (24, 48 and 72 h post inoculation). The each solid line represents a gene at three different time points. The figures are separated by experiment, control and two genotypes. The result illustrates expression differences between resistance and susceptible genotypes at 72 hpi.

## Slide 2
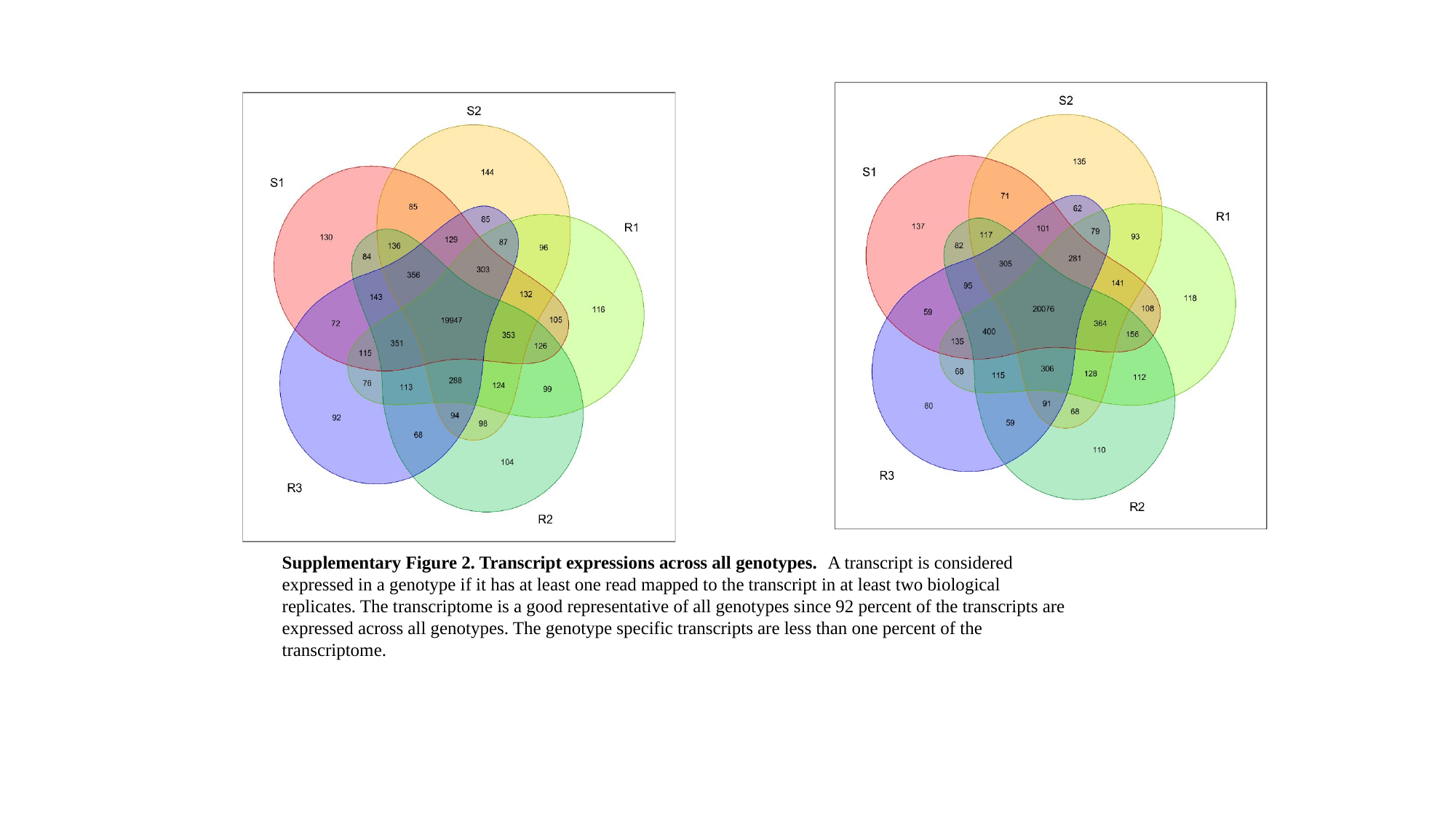

Supplementary Figure 2. Transcript expressions across all genotypes. 	A transcript is considered expressed in a genotype if it has at least one read mapped to the transcript in at least two biological replicates. The transcriptome is a good representative of all genotypes since 92 percent of the transcripts are expressed across all genotypes. The genotype specific transcripts are less than one percent of the transcriptome.

## Slide 3
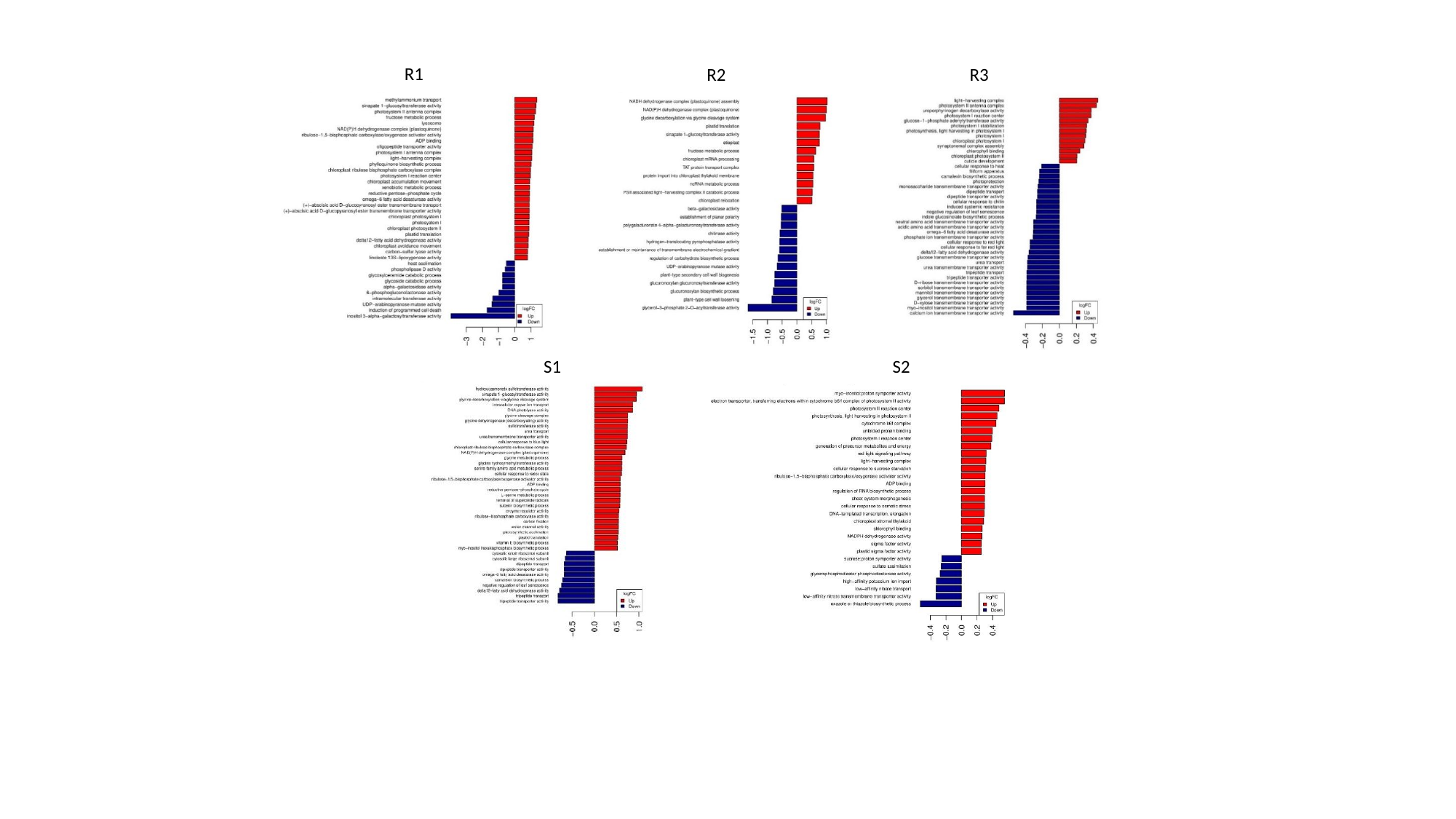

R1
R3
R2
S1
S2

## Slide 4
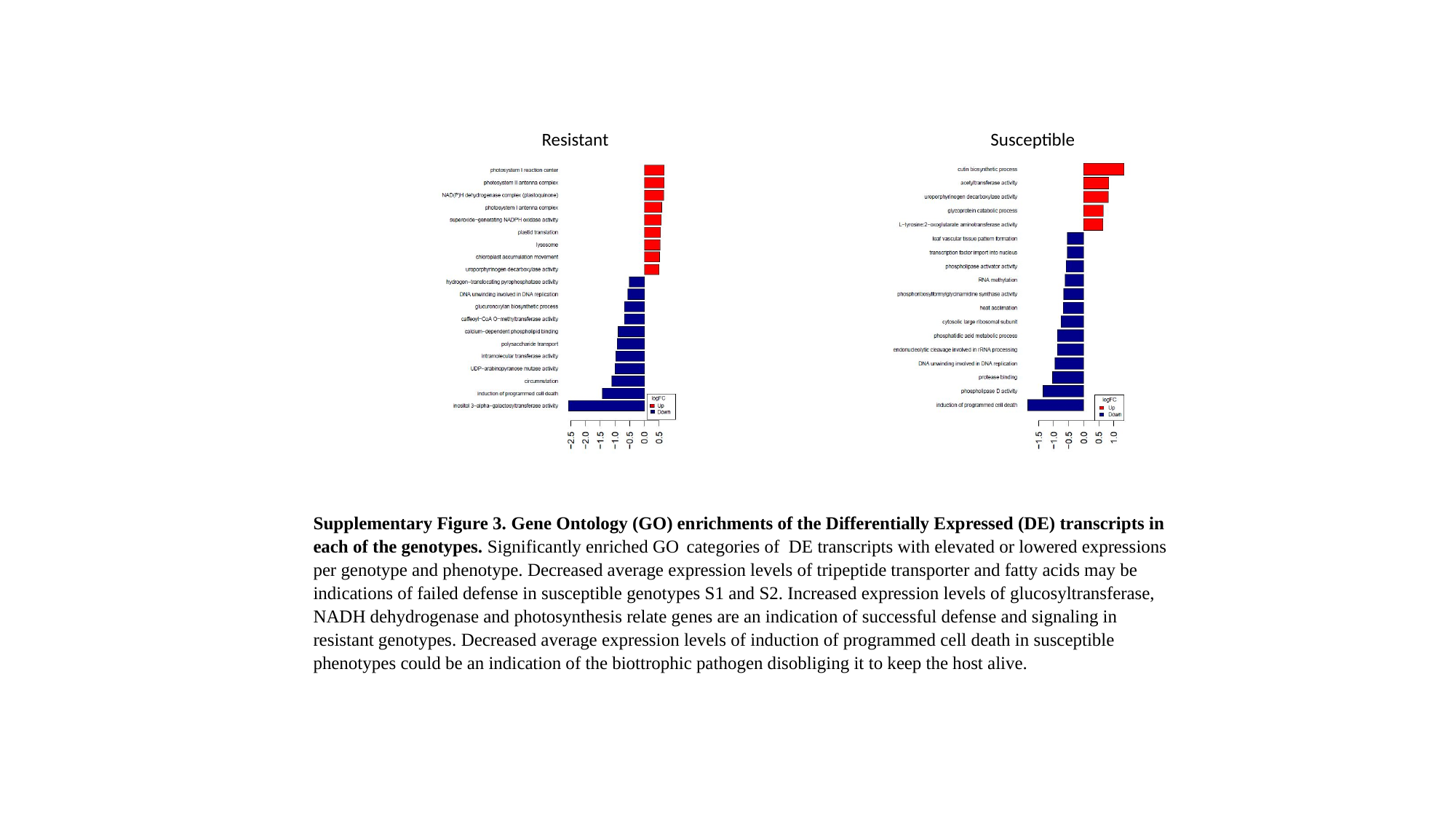

Susceptible
Resistant
Supplementary Figure 3. Gene Ontology (GO) enrichments of the Differentially Expressed (DE) transcripts in each of the genotypes. Significantly enriched GO  categories of DE transcripts with elevated or lowered expressions per genotype and phenotype. Decreased average expression levels of tripeptide transporter and fatty acids may be indications of failed defense in susceptible genotypes S1 and S2. Increased expression levels of glucosyltransferase, NADH dehydrogenase and photosynthesis relate genes are an indication of successful defense and signaling in resistant genotypes. Decreased average expression levels of induction of programmed cell death in susceptible phenotypes could be an indication of the biottrophic pathogen disobliging it to keep the host alive.

## Slide 5
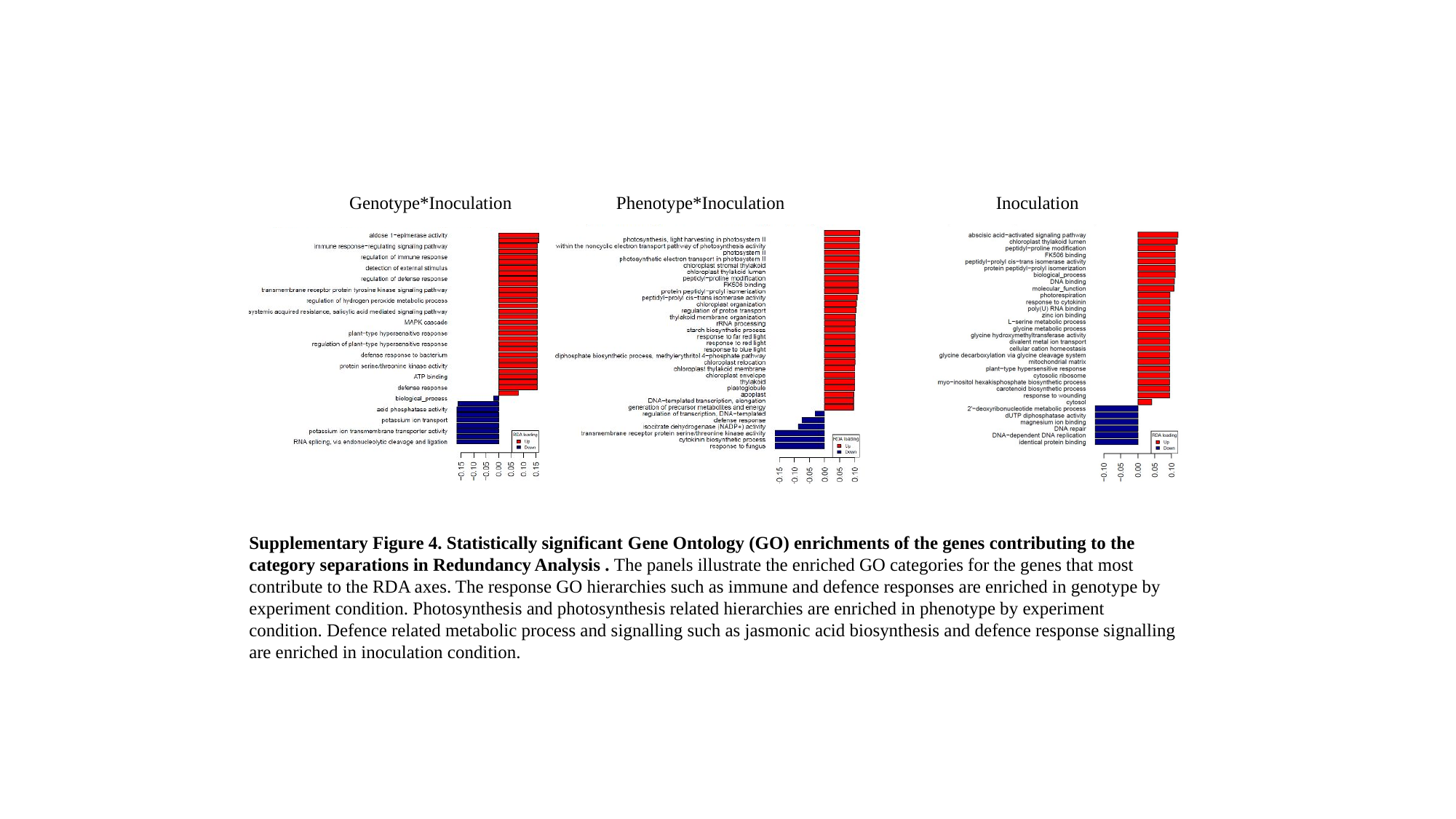

Inoculation
Genotype*Inoculation
Phenotype*Inoculation
Supplementary Figure 4. Statistically significant Gene Ontology (GO) enrichments of the genes contributing to the category separations in Redundancy Analysis . The panels illustrate the enriched GO categories for the genes that most contribute to the RDA axes. The response GO hierarchies such as immune and defence responses are enriched in genotype by experiment condition. Photosynthesis and photosynthesis related hierarchies are enriched in phenotype by experiment condition. Defence related metabolic process and signalling such as jasmonic acid biosynthesis and defence response signalling are enriched in inoculation condition.

## Slide 6
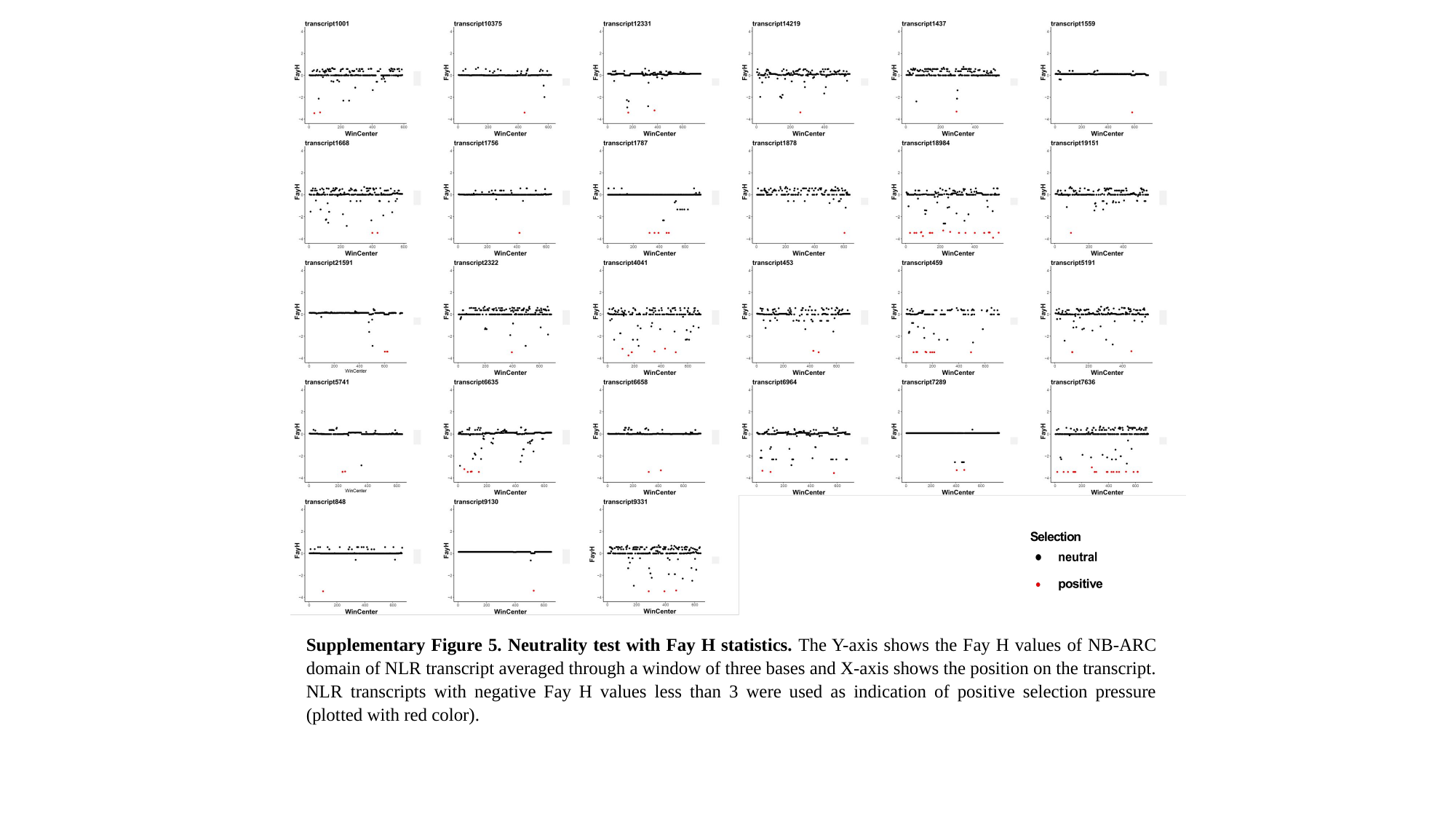

Supplementary Figure 5. Neutrality test with Fay H statistics. The Y-axis shows the Fay H values of NB-ARC domain of NLR transcript averaged through a window of three bases and X-axis shows the position on the transcript. NLR transcripts with negative Fay H values less than 3 were used as indication of positive selection pressure (plotted with red color).
