# Supplementary material for: Genotype-Specific Expression and NLR Repertoire Contribute to Phenotypic Resistance Diversity in Plantago lanceolata
Source: Front Plant Sci. 2021 Jul 12;12:675760. doi: 10.3389/fpls.2021.675760 (PMC8311189; doi:10.3389/fpls.2021.675760)
Supplement: Supplementary file 1 [file Data_Sheet_1.zip › Supplementary_File_1_qpcr.docx]

| **Supplementary File 1.** Sequences used for the pcr experiment and their Arabidopsis orthologue.   \| **Table 1. Primers used in the study.**  **Gene** \| **Name** \| **Sequence** \| **Primer efficiency** \| \| --- \| --- \| --- \| --- \| \| >>Elongation.factorCL4119.Contig1_10 \| Ref.1.F \| TGACAGACGATCTGGCAAAG \| 100.65 \| \| >>Elongation.factorCL4119.Contig1_10 \| Ref.1.R \| ACGTCCCAAAGGAGGGTACT \|  \| \| >>GAPDH28221_10 \| Ref.2.F \| AAGGTGCTGACATTCCAACC \| 98.90 \| \| >>GAPDH28221_10 \| Ref.2.R \| TCATCCATGACCTTGACGAA \|  \| \| >>Actin_34737_10 \| Ref.3.F \| GCTCCGTGTTTCTCCTGAAG \| 95.65 \| \| >>Actin_34737_10 \| Ref.3.R \| CTGGATCGCAACATACATGG \|  \| \| Unigene47041_ORF_p1_1 \| T.2_F \| CTCCCTCTGGCTCAGGTAGT \| 89.20 \| \| Unigene47041_ORF_p1_1 \| T.2_R \| CAATTGGAGCCCCACCATCT \|  \| \| Unigene36744_3_ORF_p1_1 \| T.6_F \| GCAGAAATGAGGCTGGGGAT \| 90.00 \| \| Unigene36744_3_ORF_p1_1 \| T.6_R \| CAAAGACGGCGAAAGGTTGG \|  \| \| >Unigene37535_10_ORF_m3_1 \| T.12_F \| TACCTGCCAACTTCCGCATT \| 96.45 \| \| >Unigene37535_10_ORF_m3_1 \| T.12_R \| CTGGACAGCTGATGACTGGG \|  \| \| >Unigene36964_3_ORF_p1_1 \| T.13_F \| AAGTCGAGAAATCACGCCGT \| 96.85 \| \| >Unigene36964_3_ORF_p1_1 \| T.13_R \| AGCGAACACGAGGACCAAAA \|  \| \| >CL1838.Contig15_10_ORF_p1_1 \| T.14_F \| GTACGTGTTCGCCAGATTGC \| 95.00 \| \| >CL1838.Contig15_10_ORF_p1_1 \| T.14_R \| AGCTGCATCTCATGTCTGCT \|  \| \| >Unigene48130_3_ORF_p1_1 \| T.15_F \| ACAGCAGGTCCCTATCGAGT \| 97.45 \| \| >Unigene48130_3_ORF_p1_1 \| T.15_R \| TCATGGTGTGATGCTGGCTT \|  \| \| >Unigene35676_10_ORF_p1_1 \| T.17_F \| TGTTTATCGGCTGCCCAAGT \| 92.50 \| \| >Unigene35676_10_ORF_p1_1 \| T.17_R \| AAAACTGGCTCTTCCCCTGG \|  \| |  |  |  |
| --- | --- | --- | --- | --- | --- | --- | --- | --- | --- | --- | --- | --- | --- | --- | --- | --- | --- | --- | --- | --- | --- | --- | --- | --- | --- | --- | --- | --- | --- | --- | --- | --- | --- | --- | --- | --- | --- | --- | --- | --- | --- | --- | --- | --- | --- | --- | --- | --- | --- | --- | --- | --- | --- | --- | --- | --- | --- | --- | --- | --- | --- | --- | --- | --- | --- | --- | --- | --- | --- | --- | --- | --- | --- | --- | --- | --- | --- | --- | --- | --- | --- | --- | --- | --- | --- | --- | --- |
|  |  |  |  |
|  |  |  |  |

|  |  |  |  |
| --- | --- | --- | --- |
| **Reference gene sequences:** |  |  |  |
| Elongation factor (Ref.1)  >CL4119.Contig1_10_PLANT2  ATGCACCTGGACATCGTGATTTCATCAAGAACATGATTACTGGTACCTCACAG  GCTGACTGTGCTGTTCTCATCATTGACTCCACCACTGGAGGTTTTGAAGCTGGTATCTCC  AAGGATGGTCAGACCCGTGAGCATGCATTGCTTGCTTTCACTCTTGGTGTCAAGCAAATG  ATTTGCTGCTGTAACAAGATGGACGCCACCACACCTAAGTACTCAAAGTCCAGGTACGAT  GAAATTGTCAAGGAAGTGTCCTCCTACCTAAAGAAGGTTGGATACAACCCAGACAAAATC  GCATTCGTGCCCATTTCTGGATTTGAGGGAGACAACATGATTGAAAGGTCCACAAACCTT  GACTGGTACAAGGGTCCTACCCTCCTTGAGGCTCTTGACAACATCTCTGAGCCAAAGAGG  CCATCAGACAAGCCCCTTCGTCTACCTCTTCAGGATGTCTACAAGATTGGTGGTATTGGA  ACTGTGCCAGTGGGACGTGTTGAGACTGGTGTTATCAAGCCTGGTATGGTTGTCACTTTT  GGCCCTACTGGTTTGACTACTGAGGTCAAGTCCGTTGAGATGCACCACGAGTCCATGCCA  GAAGCTCTTCCTGGTGACAATGTTGGGTTCAACGTTAAGAATGTTGCTGTCAAGGATCTG  AAGAGAGGTTACGTTGCCTCCAACTCCAAGGATGACCCTGCCAAGGAAGCTGCTAACTTC  ACTTCCCAGGTCATCATCATGAACCACCCTGGCCAGATTGGACAAGGTTATGCTCCAGTG  CTCGATTGCCACACCTCCCACATTGCTGTGAAGTTTGCTGAACTTGTCACCAAGATTGAC  AGACGATCTGGCAAAGAGCTCGAGAAGGAGCCCAAGTTCTTGAAGAATGGTGATGCCGGT  ATGGTTAAGATGATTCCAACCAAGCCCATGGTTGTGGAGACCTTCGCTGAGTACCCTCCT  TTGGGACGTTTTGCTGTGCGTGACATGAGACAAACTGTTGCTGTTGGTGTCATCAAGAAT  GTGGAGAAGAAGGACCCATCAGGTGCCAAGGTTACCAAGGCAGCTGCCAAGAAGGGAGCC  AAGTGA  GADPH (Ref.2):  >Unigene28221_10_PLANT2  ATGGCTTCACATGCAGCATTGGCTTCTTCAAGAATC  CCAACAAGCACCAAGCTTCCATCCAAGACCACTCATTCTTTCCCTGCTCAATGCTTCTCT  AAGAAGGTTGAATTGCCAGAGTTCACTGGGCTCAGGTCAAGTGGAAATCTGACTTTTGCC  AGCAATGCTGGACAGTCTTCTTTCTTTGATGCAGTGGCTTCTCAGCTTACTACCAGGGCT  GTACCATCAACACCTGCCAAGTTAGAAACTGTAGCCAAATTGAAGGTGGCCATCAACGGT  TTCGGGCGCATTGGCCGTAACTTCCTTCGCTGCTGGCATGGACGCAAGGACTCACCTCTC  GAAGTTATCGTTGTCAACGACAGTGGCGGTGTTAAAAACGCCTCTCACTTGCTGAAGTAC  GACTCCATGCTCGGTACCTTCAAGGCAGATGTTAAGATAGTCGACAATGAGACCATCAGC  GTCGATGGGAAGAATATCAAGGTGGTCTCCAGCAGGGACCCTCTCAAACTTCCATGGGCT  GAGATGGGCATTGACATCGTCATCGAGGGAACCGGTGTTTTCGTCGATGGTCCAGGAGCT  GGCAAACACATCCAAGCCGGAGCAAAGAAGGTTATCATTACTGCTCCGGCCAAAGGTGCT  GACATTCCAACCTATGTTGTTGGTGTGAATGAACAGGACTATGGTCATGATGTTGCTGAC  ATCATAAGCAATGCTTCTTGCACCACCAACTGTTTGGCTCCTTTCGTCAAGGTCATGGAT  GAGGAATTCGGTATTGTCAAGGGTACAATGACAACTACTCACTCCTACACTGGTGATCAG  AGGCTGTTGGATGCTTCACACAGGGACTTGAGGAGAGCTAGAGCTGCAGCACTGAACATT  GTGCCAACAAGCACCGGTGCAGCTAAGGCCGTGTCTCTTGTTTTGCCTCAGCTCAAGGGA  AAGCTTAATGGCATTGCTCTGCGTGTTCCAACACCTAATGTATCAGTAGTTGATCTAGTC  GTGAATGTTGAGAAGATAGGAATTACAGCTGAAGATGTAAACGCTGCGTTCAGAAAGGCA  GCTGATGGGCCATTGGCTGGTGTGTTGGCTGTCTGTGATGAGCCTCTTGTGTCTGTTGAC  TTCAGATGCACCGACGTTTCATCCACCATCGACTCCTCTTTGACAATGGTTATGGGAGGT  GATATGGTCAAGGTTGTCGCTTGGTACGATAACGAGTGGGGATACAGCCAAAGAGTTGTC  GATTTGGCTCATCTGGTGGCGAACCAGTGGCCCGGTGTGGCTGCAGCAGGAGGAAGTGGA  GACCCCTTGGAGGATTTCTGCAAGGACAATCCTGCAGATGAAGAGTGCAAAGTTTATGAA  GCCTAA |  |  |  |
| Actin (Ref.3):  >Unigene34737_10_PLANT2  ATGGCCGACGGAGAGGATATTCAGCCCCTTGTTGTGGACAATGGTACTGGAATG  GTTAAGGCTGGCTTTGCTGGTGATGACGCTCCAAGGGCGGTGTTTCCTAGCATTGTAGGT  CGTCCCCGGCATACGGGGGTCATGGTTGGTATGGGGCAGAAGGATGCTTATGTTGGAGAT  GAAGCCCAGTCGAAGAGAGGTATTCTCACCTTGAAGTATCCCATCGAGCACGGTATTGTC  AGCAATTGGGATGACATGGAGAAGATTTGGCATCACACATTCTACAATGAGCTCCGTGTT  TCTCCTGAAGAGCACCCTATTCTGCTCACTGAGGCACCTTTGAATCCTAAGGCTAACAGG  GAGAAAATGACGCAAATCATGTTTGAGACCTTTAATGCACCAGCCATGTATGTTGCGATC  CAGGCTGTCCTTTCGCTATATGCTAGTGGACGTACAACTGGTATAGTGCTCGACTCTGGT  GATGGTGTGAGCCACACTGTTCCTATCTACGAGGGTTATTCACTTCCACATGCAATCTTG  CGGTTGGACCTTGCTGGTCGTGATCTCACCGATTATCTCATGAAGATTCTGACAGAGAGA  GGTTACATGTTCACCACAACTGCAGAGCGGGAAATTGTTCGTGACGTAAAGGAGAAGCTA  GCATATGTCGCGATTGATTATGAACAAGAACTGGAAACCGCAAAGAACAGTTCTGCTGTG  GAGAAGAACTATGAGCTTCCCGATGGACAGGTTATCACCATAGGTGCTGAGAGGTTCCGA  TGCCCAGAAGTGTTGTTCCAACCATCTCTTGTAGGAATGGAATCTCCCGGAATACATGAA  ACAACCTACAACTCCATCATGAAATGTGACGTGGATATCAGGAAGGATCTCTACGGAAAC  GTTGTGCTCAGTGGTGGATCAACTATGTTCCCTGGTATTGCGGATCGTATGAGTAAGGAA  ATAACAGCTCTTGCTCCAAGTAGCATGAAAATTAAGGTTGTCGCACCACCTGAGAGGAAG  TACAGTGTGTGGATCGGAGGGTCTATCCTGGCTTCTCTCAGTACTTTCCAACAGATGTGG  ATTACAAAGGGTGAATATGATGAATCTGGCCCTTCAATTGTTCACAGGAAGTGCTTCTAA |  |  |  |
|  |  |  |  |
| **Disease induced genes tested for selecting the time point**  >Unigene47041_10_PLANT2_ORF_p1_1 **similar to AT5G53890** This is T2  ATGGAGCATCTATTCTATTTGGATTTTTCCAATAATTCTCTGACAGGAGAAATACCAAAGAGTATGACAGAGCTCAAGAGCCTCATCTCTGCGAATAGCTATGCTTCCAGTCTGAATACTTCGTCTGGCATTCCCTTTTTCATCAAGAGGAA CCAGAGTGCCAATGGTTTGCAGTACAACCAAGCTTCGAGTTTCCCGCCATCCATACTATTGAGTAATAACCGATTG AATGGATCCATTCGAGCTGAAATTGGAAAGCTGAGACAACTTCATGTCTTGGATCTTAGTAGGAATAACATATCAG GGACAATCCCAGGCTCCATCGCAGATATGGCAAATCTCGAGACATTGGATTTGTCTTACAATGATCTTCAGGGATC GATTCCTTCATCCTTCAACCGGCTCACCTTCCTATCAAAGTTCAGTGTGGCTTATAATCATCTCCAGGGAGCAATT CCGACGGGAGGTCAATTTCTTAGTTTCCCGAGCTCAAGCTTTGATGGCAATCCCGGTCTTTGTGGAAAGTTTTTTT CTCCGTGCGTTACAACTAATATGGGGCTGCAACATCCTACTCCCTCTGGCTCAGGTAGTAAGTTTGGTCGAGGAAG TATACTCGGAATCACGATCAGCATTGGAGTTGGAATTGCTCTACTCCTGGCTGTTCTTCTTCTTAGGATGTCAAGA AAAGATGGTGGGGCTCCAATTGTTGATGTGGAAGAAGATATAAGCTCTGGGCCTCGGTTTTCAGATTTTGGCCCTC CGAAGCTTGTGCTTTTCAAAAATGCAGAGTGCAAGGATCTTACCATCTCAGACATCCTTAATTCGACAAACAATTT TAACCAGTCAAATATTGTCGGATGTGGTGGATTTGGTTTGGTCTATAAGGCCGAATTACCTAATGGCAGGAAAGCT GCTATCAAGAGGCTAACTGGGGACTGTGGGCAGATGGAACGTGAATTTCATGCGGAAGTTGAAGCTCTCTCCCGAG CACAACATAAAAACCTTGTATCTCTTCAAGGGTATTGCCGTTATGGGAGTGACAGGTTGTTAATTTACGACTACAT GGAAAATGGGAGCTTGGACTATTGGTTGCACGAGAGAGTTGATGGGAGTTCATCTCTTACATGGGAAACAAGATTG AGAATTGCTCAAGGCTCAGCTCATGGGTTGGCTTATTTGCATAAAGAGCCAAATATAGTTCACCGAGACATCAAAA CAAGCAACATCCTCTTAGATGCGAAGTTCAGTGCTCATTTAGCTGATTTTGGTCTCTCTCGGTTACTTCATCCCTA TGATACTCACGTAACGACAGATTTGGTTGGTACATTGGGATATATTCCGCCAGAGTATAGTCAGACACTGACTGCA ACGTTCAGGGGTGATGTGTACAGCTTTGGCGTTGTGCTCCTGGAGCTTATTACCGGAAGAAGACCAGTTGAAGTTT GTAAAGGACGAAATTGCCGTGATTTGGTGGGTTGGGTGTACCAAAAGAAGTCTGAGATGAAGGTAGAGGAGATATT TGACTCATCAATGCAGCGAGGCAATGATTGTGAAAAGCACATCGTGCAGGTACTTGGTATAGCTTGTAAATGTATT GACCAAGATCCTAGGAGGAGACCATCAATTGATGAAGTCGTGTCATTGCTAGATGCGATCGAGATAGAAAAAGTTTGA  >Unigene36744_3_PLANT2_ORF_p1_1 **similar to AT1G53350** This is T6 ATGTGGATCGCCGAGGGTTTTATACAGCAGAAGGATGGTATCAGCTTAGAAGATACTGCAGAAAGTTATTTGGATG ATCTCATTAACAGGAACTTGCTAAGAGTTGACAAGAGAAGGCGAGATGGAAGAGTAAAGACGTGTCGTCTTCATGA TATGCTACGTGATTTTTGCAGAAATGAGGCTGGGGATAAAAGAGAAAATTTCCTCCAAGAAATGAAAAGGTCTGGA GGTTCTTTTGATCCTCCAGTTTCTAAAGTACCAACCTTTCGCCGTCTTTGTATTCATTCCAATGTCATGGGTTTTA TTTGTTCAAAACCGTATGGTCCTCGTGTTCGCTCATTTGTGTGTTTCTCCAAGGAAGAAATCAACCTACCATCAGA ACACATCTCTTCCATCCCTGCATATTTCAAGCTACTCAGAGTCTTGGAAGTCAAACCAGTCAAGTTCACCAAAATC CCGAGCGATATGTATCAACTTGTTCATATGAGGTACATAGCACTATCATTCAATTTGGCGATTCTTCCTCAAGCAT TTTCAAAGCTTTGGAATTTACAGACTCTCATCGTTGACACCACGCAAAGAACCTTAGACATCAGAGCTGATATATG GAAGATGAATCAGTTGAGACATTTGAAGACGAACGCATCTGCTACTCTGCCAAAGACAGGTAAAGGTAGCTTAGAA GGTGAAAAGATTCAAAGTCTTGGAACAATATCACCACAAAGTTGCACTGATGTGGTTTTTGAGAAGGCTCGGTATG TGAAGAAGTTGGGCATCCGTGGACGTCTAGCCATGCTCATGGATACAAAGAACGGATCATTTGATAGTTTGGCGAA GTTGGGTAATCTTGAGAAACTAAAGTTGTTGAACGACGTATTTCCTAGTCCACCTTCTGAAGGCCAACTACGCAGC CTTCCACCGCCATACCAATTCCCACCGAAACTGAGGAGCCTGGCACTGGCTGATACATTTCTCGATTGGGCTCATA TGTCTACTCTTGGATTGTTGGAGAACCTTGAGGTGCTGAAATTGAAAGATAAAGCATTCATGGGAAAATCCTGGGA GCCAGCTGATGGAGGTTTCCGTAAACTTGAAATTTTATACATTGGGCGAACAGATCTTGTGATATGGATGGCTTCA AAGCATCACTTCCCTAGGCTTAGAACACTTGAGTTGAGGAACTGTGAAGAGCTTCAGAAAGTGCCGATCGAACTTG CTGATATATCAAGCTTCCAGATGCTAGACGTGTATCGCAGTAAATTTGCTGCAGCCTCTGCCAAGGCAATTTATGA GAAGAAAAAGGAGATTAACGAAGGACAAACCACCAAATCTAACGGATTTAAGCTTACACTTTTTCCTCCTGAAGAGTGA  >Unigene37535_10_PLANT2_ORF_m3_1 **similar to AT5G35450** This is T12 ATGCGCATTGATGTGTCCTCAGCAACAATTTGTATGCGAACATTTTCTGACACTTGTTCCCACGTGTTCCTCGCTACAAC CATATCTTCTGTCGTAGAAACTTTCTTGGCAAGAATTCCTCCAATGACCACTATTGCAAGCGGTAGCCTGTCACATTTGT CAACAATGAGTCTTCCAAAACATTCCAATTCTGTGGGACACTCCGGCACACCAAAAACCTCGTATCGAAACAGCAGCC AACTTTCATCCTCCGTCAAGAATCGCAACTTGTGGGGAGCTCTACTACGGTTAGCATACCTGCCAACTTCCGCATTTCG ACTTGTGATCAAAATTTTGCCAGAATTATTATTTGCTGGCAAAGCAATTTTGAGGCTATCCCAGTCATCAGCTGTCCAG ACGTCATCCATCACGATCAAGAACTTTCCATTCTGCAAATGTTTAGCCACTTCATGGGCCAACTCTGCATCATTTTTCTT GCTTATTTCATCAGTGACCTGA  >Unigene36964_3_PLANT2_ORF_p1_1 **similar to AT1G58400** This is T13 ATGCTTCGTGATTTCTGCAAAAATGTTGCTGGAAGTGAAAGAGAAAATTTCCTCATAGAAATAAAAAAGTCCAGACAG GGAGCTTTTGATCCTCCGGTTGCTCAAGTCGAGAAATCACGCCGTCTGTGTATACATTCTAATGTTTGGAACTTTATTTC TTCAAGACCTTTTGGTCCTCGTGTTCGCTCTTTCGTTTGTTTTTCCCAGGAAGAGATCAACTTGCCATCAGAGAACATTT CTTCCATCCCTTCAGCTTTCAAACTACTCAGAGTTTTGGAAGCAAAACCCATCAGATTCAGCAAACTTCCTAGTGATATG TATCATCTTGTTCATTTGAGGTACATAGCTATGTCGTTTTGTTCAACTATTATTCCTGCAACGTTCTCGAAACTTTGGAG TATGCAAACTCTTGTTGTTGATACGACATCTCGTCTGTTAGACATTAAAGCAGACATATGGAAGATGAAACAGTTTAG GCATTTGAAGACTAATGCATCTACAATTTTGCCCAAACCAGAAAGAGGTAGTAGTAGTACTACAGAAGGTGAAAACAT TCAAACACTCGGTACAATATCGCCTATAAGCTGCACAGAGGCTGTTTTTGGAAGGGCCCGGTTCGTGAAAAAGTTGG GAATTCGTGGACGACTCTCCTTGCTTCTTGAAGGGAAGAATGGGTCATTTGATGGCTTAGGGAAGCTGAAGAATCTTG AAAAGTTGAAATTGATAAATGATGTGTTTCCGGGTTCAGCTTCTGAAGATCCATTACCCGGCCTGCCTTCACCTTACCA GTTTCCACCAAAACTAAAGAGCCTGACCTTAGTTGCTACCTTTCTTGATTGGAACAAGATGTCTATCATTGGACTTTTG GAGAACCTAGAGGTGCTCAAGTTGAAAGATAAAGCATTCGTTGGAAAAACTTGGGCAGCTATGGATGGTGGCTTTCG CAAACTTGAAGTCATGTACATTGGGCACATAGATTTAGTGATTTGGATGGCTAGTAAGCATCATTTCCCCAGGCTTAG AAGCCTTGAGTTGAAGAACTGTGAAGACCTCCAGCAACTTCCAGCTGAGCTGGCTGAGATACCTAGTTTCCAGACGCT AGAGCTGTATCGCACTAATAAGGCAGTTGCAGCTTCGGCAAGGAGTATCCGGGAGAAAAAGGTGCAGATGGAAAGT GCAGAGACTACCAAATCCGGTGGATTTAAGCTCTTGATATTTCCGCCGGATGAAATATGA  >CL1838.Contig15_10_PLANT2_ORF_p1_1 **similar to AT1G50180** This is T14 ATGACTCCCACTCACTTGGTGGGAGTGGACAAGTGTTTGATCAAACTACGTGATCGAGTCACTTCACGATCTCGTGCA CTGGAAATCATCCCCATCGTAGGCATGGGGGGAATTGGTAAGACTACTCTAGCCCGTAAACTTTACAATGAGTATTCT TCTAATCAGCACTTTGATATCTGTTTATGGGCAACCGTGTCCCATGAATATGATCAACGCCAAATTCTTCTAAGCCTTCT AGGGTGTACTAAGAGTGATAAGAAAAATGAAGAACTGAAAGTTGATGTGCACAAAAAGTTATGGTCTAGAAGATACT TGATTGTATTGGATGACGTTTGGAGTTCCAAGGTGTGGGATGATATCCGTACGTGTTCGCCAGATTGCAATAACAGGA GCCGCATCATTTTGACCACGAGGGAGAACAATTTAGCTGATTATGTTAGCTCCAGCAGACATGAGATGCAGCTACTCA ACAAGGAAGATAGTTGGGAGCTCCTCAGTAAAATTGTGCATGGTGGGGAAAATTTGCCAAATGGTTTAGAAGAAATT GTGAAAAAATGTGGAGGGCTACCTCTTGCACTTCAGGTGATTGGGGGTTTCATGTCACGAGTTGAGAATACAAAAGA TTTGTGGGAGAAAGTTGCAAATGACATAAGTTCATTCATCATAAAAGAAGACGATCAATTCTCGGGGATATTTTCTTG GAGCTATGCCAATTTGCCACATCATTTGAAGCCTTGTTTACTTTACATAGGTGCTTTCCCCGAAGATCATGACATTCAAG CTTCAAGACTAATCAAGTTGTGGGTTGCAGAGGGATTTCTGTATCAAAAGGTCGATAAAAGTCCAGAGGAAATTGCA GAGGAGTACTTAAATGACCTCATTGGCAGAAATCTTATTTTTGTCCGTGAGAGGACATTTAGTAGGCGAGTGAAATCA TACGGCATACACGATCTCTTGCGAGACCTATGCATGAAGAAAGCTGAAGAGAAGACGTTCATTTTCTCCAAAACATTG GGAGTGTTAGAAGATGCATTATTTCTCCGTCGTGTTTGTGAGTTCTTCTGA  >Unigene48130_3_PLANT2_ORF_p1_1 **similar to AT3G14470** This is T15 ATGGCTGCAAACAAATTTTATCTAGCGTCAAATGCACTCCTCCCTCTACTGATGTTTGGACTCCTAGAATTTTGGAAAG GGAGGTGCTTGGAAACTGGAATCCAGACATGGAAGGAAACTGGAATCTTTGTCCAGGAGCCACTTCAAAAGCCTATC ACCGTAGACCGACTCATGATCATACTTCAAGTGATTCTGTTTTGGTTGATGTGGAGGACTTTTAACCTAAAGGAATCTG GAACCCATTTGGAGAAGCCATTTGATATTTTTTGGGAGGTTATCCGTTTGTTGCTTGCAGATGCCAAGGAGCAGCACA TAACCGAGGCATCATTACCACAATTATTGGAGGAGCTCGAGCTTTTGGTTGGTGCCTTGGATGATTCATGTTGCATCAT ACTATCTCCATCCAAAATGTCAGTATCACATGGGATTGGGTATGACATCATGAGTAGGTTTCCGGAGTTCATGCGTTCA CCTAGATCCAAAGTCGATGCGGGACAAAGGGTGGAATGTAACACATCAGTAAAGTGGGTGCTATGTCCTTCTTATATG GTCACAGAACCATTTGAATGCGTATCAACTGATTACTATACAGCAGGTCCCTATCGAGTTTTCCCCATAGTGCGTGGAT CAGATGCCTTTCAGAAGCTAACTATGACCAAGATAGGTGAAATCCTAGAGAAGAACACTATGTTAAGCCAGCATCACA CCATGATTCATCTTATAACTAGAGCCTATGCGACAGATGAAGATAAGAAACCTCTTACTTCAATTGTGAAAGAGCTTCA GGTTTCTGGGAATGATGAAGATAAAGAAGCTCGTACTTCTCTGTTAAGTGAGATTGAGGTGCATGAGAGAGATGAAG ATAAAGTTGTCACTTCTTTGATGAATGAGTTTCCATATCATCACCGTATGAATGAAGATAAAGTTGTATTCTCGTTGTCA AGTGGATCTCGAATTTATGGGAGAGATGAAGATAAAGAAGCAATAGTCAAAATGTTGCTTGGCAATGAGCTGTGCAG CAATGATGTTTCTGTCATTCCAGTTGTTGGACCTGGTGGTATTGGCAAAACCACACTAGTGGAACTTGTGTATGACGAC AGAAGTGTTACCGATTACTTCCATTTGAAGGCCTGGGTCAGTGTTCGTGAAGAGTTGGATGTATTTATGGTCACCAGC ACAGTTTTTGAAGCAATGACCAGCCAGAGACCCGAGTCCATGGATCTGAATGCGCTTCAGTTAAGTCTAAAGGAGCAA GTATCGCGAAGCAAGTTTCTTCTTGTTTTAGATGATGTTTTGAATGTGAATCGTGAACAATGGGATGTTCTTTATCATCC GTTCCAGTTTGGACTACCTGGAAGCAGATTTATTGTCATAACTCGTAACAATGCCGTTGCATCTATGGTGTGTTCTGCT CGATTAGTAGCTTACCACATGAAACTGTTGACAGATGATGATTGTCTAGCTATATTGTCTCAACATTCCTTTAGATCTTT CAGTGGAACTTCAAAATTTGAAGAAGTTGGGAAAGGACTATCCAAGAGATGCAAAGGCTTGCCCTTGGCAACGAAGG TGCTTGGCAGCCTGCTACGATACAAGGAAGATAAAGAAGAGTGGGATGATTTGCTGAATAGCAATGTGTGGAATTTC CATGAAGAAAATGATATTCTTCCTGTTTTGAGGTTAAGCTATCTCCATCTCCCCCCACATCTCAAGTATCTGTTTGCCTTT TGCTCGCTGTTACCAAAGGGCTGTGAATTAGACACGAATGATATGTTAAGTCTGTGGATTCGAGAAGGTTTGCTGGAT GATGCAGACGGGAGGACCTTGAAAGAAAGGAAGAGTTAG  >Unigene35676_10_PLANT2_ORF_p1_1 **similar to AT5G43470** This is T17 ATGGCAGCTTATGCAGCTCTACTCTCTCTCAACTTCACCATCGACCAGCATCACCACCGCTCTGTTGTTTACCCACCTCA ATACCCGATTCAGTCTGCAGTCAAAGAAACAATCACTTTTCTTCTCAAAGTTTTCCAGAATTACGATTACCAAATGATCC AGCAACAAGTTGTTGAGGTGGAGAGAGAGATAGCGGAGGCAGCTTATGATGTTGACTATGTCATTGAATGCCTCGCT TTGGATCCGGTACTTGTCAAATCTGGATACAAACAGGGGAAACACAGCTCCAAGCTTTTCTCCGAAGATGAAATGTTG CAAATAATGATGAAGTTCGTGTCTCTCAAGGAGAAGGCCATGGAGATGATGGGTGTCAAAGTCGTCACTTCTGAAGG TGATCATCAAATGCCAACAAGTCGCGCTCCAAGTGCAACTAATGCATTATACAGAATGGTGGGATTTAATGAGCAAAT GAATGAGGTCATAAATCCATTATTCGATTATGGCACAAAAAGTCTGAAGGTAATCCCGATTGTTGGAAAGGGCGGCAT AGGTAAGACTACTCTTATTCGAAATGTTTTTAATTATGAACGCTCCGTGAATTATTTTAAAATTAGTATATGGTGTACAA TCTCTCAGTCACCCGACCTCCAAAGTGTACTCTCAAGTTTTCTACAAGACCTTGGGATTGTTGATGATGGCCAAACTAA TCCAGGGGAACGATTGCACAAGCGTTTGTATGGTAAGAGTTACCTTATAGTGCTAGATGATATATGGGGCGTTGATGT TTGGGATACAATCAAAAGTTTCTTTCCGGATAATGGTAATAAGAGTCGGATCATTATTACCACAAGGATGTTATCTGTG GCTTCTTATATTGGAACTTGCGAACTATATGAATTACGATTATTAGACAAAGAGGCAAGCTGGGATCTACTCAGGCAA AAGGTGTTTGCAGGAGATGTATGTCCTCCTGAGTTGGAGCATATCGGGATGCAGATCGCCCGAGGTTGCAGGGGACT ACCTTTAGTGATTGTTGTCATTGCGGGGCAACTGATGACGAACAATATGACAAGAGATTCCTGGGAAAGTGTTGCAAT GCGTTTGAGATCAGCCGTAGATTCTGAAGGCCAAGATTGGTATCTACAGATTCTGTCTTATTGTTATGAAAACTTGCCT GTTCATCTTAAACCCTGTTTATTATACATGGGAGTTTTTCCGGAGGATCATAGCATTCAAGTCTCTCTACTCATTAACAT GTGGATTGCGGAGGGATTTTTGAAACCCAATCGAGATCAATGCTTAGAAGACGTTGCCATTGAGTATCTCGTGGATCT GGTCCAAAGAAATCTCATTTTGGTTACTGAGCTATCCTCCAGTGGGAAGATTAAATATCTCCAAATTCATGATTTCATA AGGGATTTGTGCCTAATGAAAGCTCATGAATATAACTTTCTTCGTAGCCGCACATACCTTAACCTCGGCATTCTTCCTG GCAAAGAAAGAGATTATCCTCTGCACGTACTCCCCGCGCGAGAGAGGTTTATAAGAGTATCTCCTTTCAAACTGGAAA GTAAATGGGATTTTTGTGCTAATAAGAACCTGATGCACTTTCGCCTCCGGTGTATTTGTTTATCGGCTGCCCAAGTTAA GGAAATTTTGATTTGTTCAGCATCTCTTGCGTCGATATCCATGTTTCAAAATCTACACAGTCTAACTATGAAGACTGTTC CAGGGGAAGAGCCAGTTTTCCTTCCACCTCAGATATGGGAGATGCCACTGCTTAAACATGTAGAGATTGAACGAGTTG TCTTACCTAATCCTCCAGGCGCCGAATCCTATGTCGATGGGAGAGAGCCTTACGTGCTCATGAATATGCTGACACTGCT CAATGTGTACAATTTCAAGTTCAGTGACGAGGTTGTCGATATGATTCCAAATCTGAAGAAAATAAAAATATCATACAC AGAAGAGTCTGAAAATGTGGAAATGTCAAGCTACTGTCTCAATAATCTTGCCCAGCTATATAAACTTGAATCTTTAAGT TGCTGTGTGAAAGGAAAGATTTCTATAGAACACATAGATTTCCCGCAACGGCTGAGAAGTTTGACATTGATTGGTGGC GTGATTCCTTGGAAGGACATGTATAAAGTGGGCTGGCTCCGTAAGCTTGAAAAGCTTTCTCTGTGGGATGATGCCTTC TGCAGTAGTAAGTGGACTGTGTATGAAGGCCAATTCTATGAGCTGAAGGTCTTAAAACTTGGATGCTCAACTTTGGAG CATTGGGAAACCGAAAGCAGCCACTTCCCAGCACTTGAGCGCCTGACCTGTTTTGACATGCAGCGGTTGAAGGAAATT CCAAAAGAATTTGGAAAAATTATGACTCTCCAATTGGTCTCCGTGGAGCGGTGTTGCACTTCCCTTGTTAAATCAGCAT ACAAAGTGAAAGAAGAGCAACAACAGAACCGAAATGTTTATTTTCGACTGCATTGCGACAGAGCACAGCCAGAGGAT GAGGAAGATCCGGATGACAATGATGATTCGGAATGA |  |  |  |
|  |  |  |  |
|  |  |  |  |
|  |  |  |  |
|  |  |  |  |
|  |  |  |  |
|  |  |  |  |
|  |  |  |  |
|  |  |  |  |
|  |  |  |  |
|  |  |  |  |
|  |  |  |  |
|  |  |  |  |
